# Trajectories of changes in oxytocin and vasopressin before, during, and after mother-infant interaction: a descriptive study of mothers and infants affected by postpartum depression

**DOI:** 10.3389/fpsyt.2025.1636616

**Published:** 2026-01-05

**Authors:** Tyler Harrison, Amy S. Tao, Phuonglan Vo, Soeun Kim, Sohye Kim

**Affiliations:** 1 Applied Statistics and Data Science Program, Azusa Pacific University, Azusa, CA, United States; 2 Department of Obstetrics & Gynecology, University of Texas Southwestern Medical Center, Dallas, TX, United States; 3 Texas A&M College of Medicine, Bryan, TX, United States; 4 Eunice Kennedy Shriver Center, University of Massachusetts Chan Medical School, Worcester, MA, United States; 5 Departments of Psychiatry & Behavioral Sciences, Pediatrics, and Obstetrics & Gynecology, University of Massachusetts Chan Medical School, Worcester, MA, United States

**Keywords:** postpartum depression, oxytocin, vasopressin, maternal, infant, intergenerational

## Abstract

**Background:**

Postpartum depression (PPD) is a major public health problem with significant adverse consequences for maternal mental health and infant development. Despite this, there exists surprisingly little data concerning neurobiological underpinnings of PPD. The present study examined oxytocin (OT) and vasopressin (VP), two hormones critical for mood regulation and social/maternal behavior, to provide descriptive data regarding the role of the two hormones in PPD and intergenerational risk.

**Methods:**

Twelve postpartum mothers (5 with PPD and 7 without PPD) and their infants participated in the study. Following a baseline blood and saliva sample collection, mothers and infants engaged in a 15-min semi-structured play interaction. Blood samples were collected from mothers at 5, 10, 15, and 40min following the start of the interaction, and saliva samples were collected from mothers and infants at 20min following the start of the interaction. We documented trajectories of changes in OT and VP before, during, and after the interaction, as well as associations between mothers’ and infants’ OT and VP.

**Results:**

We observed sample-wide increases in maternal OT at 5, 10, 15, and 20min, and decreases in maternal VP at 15min. Infant OT was increased at 20min. Mothers with PPD showed a more gradual increase in OT during the interaction compared to mothers without PPD. Following the interaction, OT decreased and reached its pre-interaction baseline level in mothers without PPD, but such return to baseline was not observed in mothers with PPD. Maternal and infant hormone levels generally showed positive associations. The positive association was more pronounced in OT than VP, and in the non-PPD than PPD group. OT and VP generally showed negative associations. Our data further illustrated potential inter-relationships among PPD, OT, and several important maternal characteristics.

**Conclusion:**

Our findings illustrate trajectories of the mother’s and infant’s OT and VP before, during, and after interactions, and alterations of these trajectories in the presence of PPD. The findings add valuable data to the field’s understanding of how the OT and VP systems function in healthy postpartum mothers and infants and how they become dysregulated in the presence of PPD.

## 1 Introduction

Postpartum depression (PPD), the most common complication of childbirth, is a major public health concern (1, 2). PPD involves symptoms of depressed mood, anhedonia, irritability, disturbances in sleep/appetite, feelings of worthlessness or guilt, and/or thoughts of self-harm, and is highly comorbid with anxiety (3, 4). In the United States, a Centers for Disease Control and Prevention (CDC) analysis of 2018 data found that approximately 13% of women with recent live births self-reported symptoms of PPD. These estimates rise in racial and ethnic minority groups, such as African Americans and American Indians (1), or in low- or middle-income countries (5).

In addition to the consequences for mental health in mothers, PPD also presents potential complications for their children. PPD has been shown to adversely affect mother-infant relationship (6). Indeed, some studies have found PPD to be associated with impaired maternal-fetal attachment and bonding (7, 8) and have found depression at 1 and 6 months postpartum to be predictive of bonding failure at 1 year after birth (9). Not surprisingly, children of depressed mothers are often at an increased risk for numerous adverse developmental outcomes, including neurocognitive deficits, maladaptive social behaviors, and medical and psychiatric disorders (10–12). Compared to children of non-depressed mothers, children of depressed mothers are three times more likely to experience depression later in life (13). Furthermore, children exposed to the mother’s PPD in their infancy show a striking 5-fold increased odds of developing depression by late adolescence (14). Despite the prevalence of PPD and the striking consequences PPD can have for both mothers and their infants, pharmacologic treatment of mothers during the postpartum is often hampered by concerns regarding potential effects on the breastfeeding infant. Thus, PPD is frequently left undiagnosed and is left untreated in three-quarters of diagnosed women (15).

### 1.1 Oxytocin and PPD

Despite the public health burden and barriers to treatment, there exists surprisingly little data concerning neurobiological underpinnings of PPD that could guide efforts for developing novel targets for prevention and intervention. In this context, oxytocin (OT), a neuropeptide hormone that critically supports behavioral adaptation in early motherhood and is altered in depression, has emerged as a possible avenue for treatment (16–18). OT is synthesized in the paraventricular (PVN) and supraoptic nuclei of the hypothalamus and released into the bloodstream from the posterior pituitary. Apart from its presence in the hypothalamus, OT is also found in several other brain regions (e.g., bed nucleus of the stria terminalis [BNST], central and medial nuclei of the amygdala [CMA], septum, ventral tegmental area [VTA]) known to be critical for regulation of mood/stress and expression of maternal behavior (19). Although OT has long been recognized for its peripheral actions, including uterine contraction in childbirth and milk ejection during breastfeeding, it is now viewed as a key neuroregulator implicated in mood/stress-related disorders on one hand and maternal behavior on the other (19, 20).

#### 1.1.1 OT and depression

Substantial evidence from animal models supports the stress-attenuating and anti-depressive properties of OT (20). Intraventricular and intraperitoneal injections of OT decrease depressive-like behaviors in mice (21, 22), whereas these effects are blocked by an OT receptor antagonist and are absent in OT receptor knockout mice (23). Other core or associated features of depression, including anhedonia, anxiety, sexual dysfunction, and sleep disturbance, have also been shown to be reversible following administrations of OT (16). The literature documenting OT abnormalities in human depressed patients is more limited but suggestive. Peripheral OT levels are reduced in patients with unipolar and bipolar depression, especially in female patients (24), and OT levels demonstrate an inverse relationship with depressive symptoms in major depression (25), obsessive compulsive disorder (26), and fibromyalgia (27). Studies investigating an administration of synthetic OT as a potential line of treatment have also demonstrated some promise. In an open trial study, patients with medication-resistant depression (non-responsive to serotonin-selective reuptake inhibitor [SSRI] treatment) showed a reduction in depression scores when intranasal OT was used as an adjutant (28).

While breastfeeding difficulties commonly reported in PPD have pointed to underlying OT abnormalities ((16–18); also see (29) for a review of the influence of reproductive hormones on PPD)), the field’s understanding of OT functions in PPD still remains incomplete. Extant data point to reduced prenatal and postpartum OT levels in mothers who subsequently develop PPD (30–33). The reduced OT levels predicted concurrent and subsequent PPD symptoms and were inversely correlated with symptom severity (30–33). However, findings related to synthetic OT administration and its effect on symptoms of PPD have been conflicting. Studies by Gu et al. (34) and Kroll-Desrosiers et al. (35) found that intravenous and/or intramuscular administrations of synthetic OT during labor/delivery and the postpartum were associated with greater symptoms of anxiety and depression in the postpartum, while a study by Takács et al. (36) found that such administrations lowered the risk for PPD. Similarly diverging findings have been reported in studies examining intranasal administrations of OT. Donadon et al. (37) reported that intranasal OT administration reduced negative thoughts in mothers with PPD, while work by Lindley Baron-Cohen et al. (38, 39) suggested that such positive effects may be more nuanced and potentially moderated by PPD severity. Lindley Baron-Cohen’s work (39) suggested that intranasal administration of OT increased OT levels in the mother’s breastmilk, but this effect was less prominent in mothers with PPD. Similarly, the OT administration was shown to improve mood in postpartum mothers with moderately low mood, while this effect was not present in mothers with elevated PPD symptoms (38).

#### 1.1.2 OT and maternal behavior

Research across mammalian species supports the central role of OT in the onset and maintenance of maternal behavior. Intraventricular injections of OT induce a full range of maternal behavior in virgin rats, which are normally aversive toward rat pups (40), whereas infusions of an OT antagonist into the VTA block the emergence of maternal behavior in parturient rat dams (41). These results are in line with reports documenting impaired maternal behavior in female OT knockout mice (42) and in parturient mutant mice with reduced OT neurons in the PVN (43). OT receptor densities in the CMA and BNST have been correlated with the quality of maternal care in rats (44). Similarly, in humans, quality provision of maternal care is dependent on the mother’s OT functioning. The mother’s OT system undergoes systematic upregulation during pregnancy (45). This rise in OT production primes the mother to provide optimal care for the child (46), and postpartum interactions with the child further stimulate maternal OT release (47, 48).

Our own work, as well as that of others, have shown that mothers with impaired caregiving show less of a rise in OT during pregnancy (49), reduced baseline OT during the postpartum period (46), and blunted OT release during mother-child interaction (48, 50, 51). We and others have, in turn, linked these abnormalities to diminished activation of OT-associated brain regions (50, 52). Although these studies have started to extend the findings from animal models to human mothers, this line of inquiry is only now beginning to be extended to a clinical sample of high-risk mothers (39), such as depressed mothers, for whom the study of and intervention in the quality of maternal care is particularly important.

### 1.2 Vasopressin and PPD

Another hormone which has received some attention in terms of its relationship to PPD is vasopressin (VP). Similar to OT, it is primarily synthesized in the supraoptic nuclei and PVN of the hypothalamus and has been implicated in the regulation of social behaviors, particularly stress and depressive disorders (53). The existing body of literature has largely characterized the activities of OT and VP as being inverse to one another, with antidepressive and anxiolytic effects being attributed to OT and depressive and anxiogenic effects being attributed to VP (20, 54, 55). However, recently a more complex relationship has been elucidated between the two hormones, particularly in the potential for OT and VP to activate each other’s canonical receptors, a phenomenon known as crosstalk (56, 57). Despite the potential role of VP in mood regulation and maternal behavior, as well as its potential crosstalk with the OT system, there has been a paucity of research examining the relationship between VP and PPD. Recent evidence from animal models have pointed to potential associations between decreased OT functions, increased VP functions, and impaired maternal behavior (58). To our knowledge, the only study to date that has addressed the association in human mothers is that from Iran by Kashkouli et al. (59), who reported a positive association between plasma VP levels and PPD symptom severity.

### 1.3 OT and VP: synthesizing what is known and what remains to be studied

#### 1.3.1 Intergenerational risk and effects on infant development

The research examined above documenting that OT (and possibly VP) mediates the process through which compromised maternal caregiving alters the child’s development is particularly relevant to PPD. Animal studies have provided a fine-grained examination of the epigenetic mechanisms responsible for the intergenerational effects. Suboptimal maternal care, which characterizes mothers with low density OT receptors and low OT synthesis, compromises the development of the OT system in the offspring, who demonstrates low OT receptor densities and activity profiles that parallel those of mothers (60). Cross-fostering and methylation studies have elucidated that these intergenerational links are contingent upon the epigenetic regulation of gene expression, mediated by the quality of maternal care during the offspring’s early life (61, 62). Efforts to extend these findings to humans are ongoing. Available data now link suboptimal mother-child interactions to the child’s reduced baseline OT, elevated stress reactivity, and blunted OT release during interactions with the mother (47, 63–65). This line of inquiry is only now starting to be extended to clinical samples of high-risk mothers, such as depressed mothers, and their infants (65). To our knowledge, VP has never been examined with regard to its intergenerational risk in human mothers and infants.

#### 1.3.2 Moderation by maternal and infant characteristics

Interest in OT as a potential therapeutic for neuropsychiatric disorders has contributed to a growth of human studies examining effects of exogenous/synthetic OT administration (66, 67). Many of these studies used intranasally administered OT to investigate whether enhancing levels of OT via exogenous OT administration would decrease neuropsychiatric symptoms and increase socioemotional functions. Despite the initial optimism around the potential of intranasal OT as a therapeutic, results that have emerged over time are mixed and inconclusive, suggesting a more complicated relationship between OT and socioemotional functions (68, 69). The inconclusive findings have been at least partly attributed to a lack of careful examination of moderating factors, including sex, age, reproductive status, medication, trauma history, and patterns of attachment. A large subset of intranasal OT studies has focused on men in efforts to minimize confounding effects of female reproductive hormones on OT and VP systems. More studies are needed that focus on women in general, and perinatal women specifically, to allow the field to tease apart the role of potential moderators associated with sex. The individual’s history of trauma and resulting attachment patterns have also received increasing attention as critical factors modifying functions of OT (and potentially VP) systems (68, 69). Trauma and attachment often arise from the individual’s early social environment, the same context where early epigenetic programming of the OT (and potentially VP) systems take place (70). Therefore, trauma and/or attachment-related variables may be associated with alterations in OT and VP receptor density, function, or affinity that may have occurred during the developmental process and remain as important moderators to be understood (71, 72).

#### 1.3.3 Measuring temporal patterns of changes through peripheral concentrations

Unlike studies on exogenous OT which have largely been inconclusive to date as described above, a growing number of studies reporting on endogenous OT levels generally converge to reveal an inverse relationship between OT levels and clinical symptoms, including symptoms of PPD (73). The growing body of literature has underscored the field’s challenges in measuring and comparing endogenous OT levels across studies. Absolute measurements of OT can vary widely across individuals, which along with the challenges introduced by the short half-life of OT and the timing of measurements in relation to its pulsatile pattern of release (74), make it difficult for the field to come to a unified understanding of when/how OT is expected to rise during social interactions, when/how it returns to baseline following the interaction, whether/how these patterns vary depending on types of peripheral samples (plasma vs. salivary vs. urine) from which OT is obtained, and how these patterns become altered in high-risk individuals/dyads. In relation to this, the field is increasingly recognizing the benefits of taking repeated measurements across time – before and after an experimental paradigm/manipulation (75) and/or with pre-determined intervals between measurements (17, 31, 33). Data that help us understand temporal patterns of changes in VP are even more sparse than those on OT. Studies that conduct serial sampling of these hormones following an experimental paradigm that are known to clearly manipulate OT and VP systems are critical in advancing the field’s fundamental knowledge of how these systems function in healthy individuals and become altered in at-risk individuals. Addressing this scientific gap has the potential to bring the field closer to using OT and VP measurements for clinical benefit.

### 1.4 Current study

The present study is among the first to explore the role of OT and VP in PPD and their potential effects on intergenerational risk. We conducted serial measurements of OT and VP in both the mother and the infant before, during, and after our semi-structured mother-infant interaction procedure to systematically document changes in OT and VP in response to the interaction. Given the paucity of research systematically tracking temporal trajectories of changes in OT and VP in mothers and infants following a social paradigm, as well as the paucity of data comparing such trajectories in healthy/low-risk vs. high-risk dyads, our aim was to provide exploratory data that will inform and guide the field in designing and implementing future studies.

We predicted that non-depressed mothers and their infants will show an expected pattern of initial rise in OT upon the start of the interaction, followed by an eventual return to baseline upon the conclusion of the interaction. We aimed to specifically track and provide descriptive data concerning temporal patterns of this rise and return to baseline. In comparison to this pattern seen in non-depressed mothers/infants, we predicted that depressed mothers and their infants will show a more compromised/moderated rise in OT during the interaction and alterations in patterns of OT’s return to baseline upon the conclusion of the interaction. Due to insufficient data reporting on VP functions in either healthy/low-risk or high-risk mother-infant dyads, our study of VP was designed to be exploratory in nature. Informed by the growing literature on the importance of measuring and examining factors that may moderate OT functioning and its associations with socioemotional outcomes, the current study was designed to assess maternal and infant characteristics that have been proposed in the literature as potential moderators (e.g., maternal history of trauma, current patterns of attachment, etc.) and to explore their potential interrelationships with OT functions and maternal PPD.

## 2 Materials and methods

### 2.1 Participants

Twelve postpartum mothers, aged 25 to 38 (*M* = 31.3 ± 4.1) years, and their infants (4 boys; 8 girls), aged 7 to 24 (*M* = 13.8 ± 5.1) weeks, were recruited through prenatal clinics, reproductive psychiatry clinics, and community advertisement in Houston, TX. Seven mother-infant dyads were enrolled into the non-PPD group and 5 dyads were enrolled into the PPD group. Inclusion criteria for the PPD group included: (a) presence of clinically significant symptoms of PPD in the mother, as evidenced by the Edinburgh Postnatal Depression Scale (EPDS) (76) score of ≥ 10 and the Center for Epidemiological Scale for Depression-Revised (CESD-R) (77, 78) score of ≥ 16; and (b) absence of current bipolar or psychotic disorders in the mother, as ascertained by the Mini International Neuropsychiatric Interview 7.0 (MINI) (79–82). Current psychiatric treatment was not an exclusion for the PPD group as long as the mother met the EPDS and CESD-R criteria at the time of the study visit. This aligns with our understanding that what affects the dyad’s postpartum experience is the mother’s symptoms and the resulting impairment, regardless of the mother’s treatment status. Inclusion criteria for the non-PPD group included: (a) absence of PPD symptoms in the mother, as evidenced by the EPDS score of < 10 and the CESD-R score of < 16; and (b) absence of current or past DSM-5 Axis I disorder in the mother, as ascertained by the MINI. Potential participants were excluded from both groups if any of the following criteria were met: (a) preterm birth < 35 weeks of gestation; (b) medical complications in the infant requiring level III or IV neonatal intensive care units (NICU) stay exceeding 48 hours; and (c) maternal substance abuse within the past year or substance dependence within the past 5 years, as ascertained by the MINI. Ethics approval for this study was granted by the institutional review board at Baylor College of Medicine. Mothers provided written informed consent in accordance with the Declaration of Helsinki.

### 2.2 Study procedure

**Figure 1:**
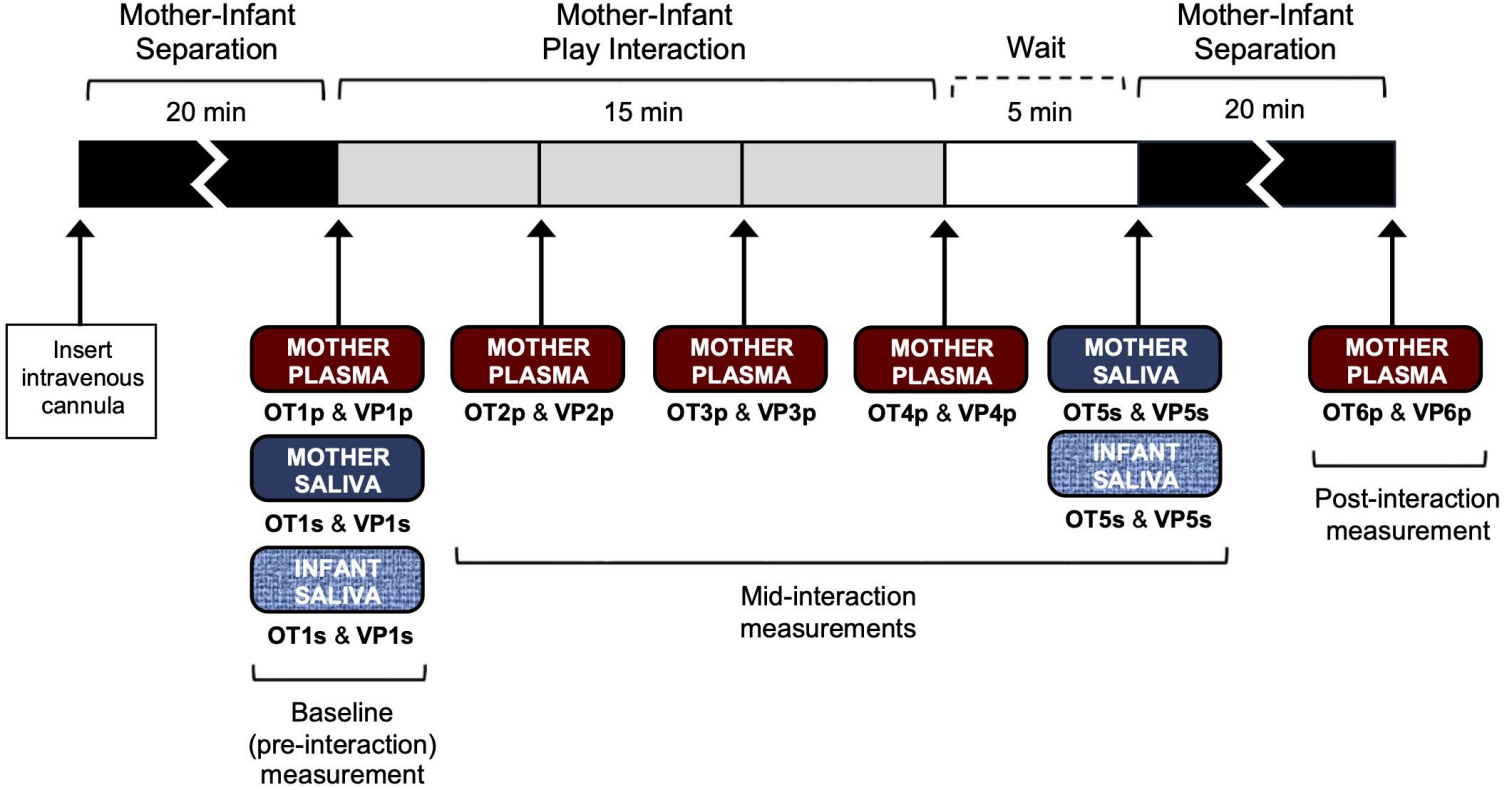
Semi-structured mother-infant interaction procedure and sample collection.

Prior to arriving for the scheduled study visit, mothers were instructed to abstain from a major meal for 1 hour, caffeine for 2–3 hours, brushing teeth for 30 min, and dental work for 24 hours. Upon arrival, mothers rinsed their mouth with water to remove food residue in preparation for saliva collection. No sample collection took place within 30 min of infant feeding. The dyads participated in a semi-structured mother–infant interaction procedure (48, 51), during which six serial measurements of OT and VP were obtained from the mother (plasma and salivary) and infant (salivary) as illustrated in **Figure 1**.

#### 2.2.1 Semi-structured mother-infant interaction procedure

##### 2.2.1.1 Baseline (20-Min Pre-Interaction Separation)

At the outset of the study visit, an intravenous cannula was inserted into the mother’s non-dominant forearm by a research nurse. This was followed by a 20-min mother-infant separation, during which the mother and infant remained in the same room without any direct contact taking place between the two. The mother completed questionnaires, while the research staff monitored the infant. Following the 20-min separation, the baseline blood and saliva sample collection took place. To minimize the possibility of the stress of sample collection confounding OT and/or VP levels, samples were collected in the following order: maternal saliva (OT1_s_ and VP1_s_), maternal plasma (OT1_p_ and VP1_p_), and infant saliva (OT1_s_ and VP1_s_).

##### 2.2.1.2 15-Min Free-Play Interaction

Following the baseline (pre-interaction) sample collection, the mother and infant were reunited for a 15-min semi-structured play interaction, which allowed the mother to first engage with their infant face-to-face, followed by a period of free-play with the infant on their lap. For the first 10 min, the mother sat on a chair and the infant was seated in a bouncy seat, which was mounted on a table and placed directly across from the mother. This setup allowed the mother and infant to sit close enough to touch each other and directly view each other face to face. The mother was instructed to play with the infant as she normally would for 10 min. After the 10-min face-to-face interaction, the mother was instructed to move the infant from the bouncy seat onto her lap and continue playing for another 5 min. At 5, 10, and 15 min into the play interaction, blood samples were drawn from the mother through the previously inserted cannula. To minimize the possibility of sample collection interrupting the ongoing play, the mother was instructed via intercom to position her arm toward a research nurse through a curtain at 5 min (OT2_p_ and VP2_p_), 10 min (OT3_p_ and VP3_p_), and 15 min (OT4_p_ and VP4_p_) into the play. Blood draw was completed by a research nurse who was seated behind the curtain.

##### 2.2.1.3 5-Min Wait Period

Upon conclusion of the 15-min play and blood sample collection, there was a wait period of 5 min before saliva samples were collected from the mother and infant. The wait period was built into our study design to allow time for changes in OT and VP levels to be reflected in peripheral samples. No direct mother-infant contact took place during this wait period. Mothers completed questionnaires while the research staff monitored the infant in the same room. To minimize the possibility of the infant sample collection confounding the mother’s OT and/or VP levels, maternal saliva samples (OT5_s_ and VP5_s_) were collected first, followed by infant saliva samples (OT5_s_ and VP5_s_).

##### 2.2.1.4 20-Min Post-Interaction Separation

Following the wait period and saliva sample collection, there was a 20-min separation period before the final post-interaction blood sample was collected from the mother (OT6_p_ and VP6_p_). Since this timepoint (approximately 40 min following the onset of play interaction) generally reflects hormones’ return to baseline levels following the initial rise in hormones during the interaction (48, 50, 51), this separation period was built in to minimize further contact between the mother and infant from confounding the mother’s OT and VP levels. As described before, mothers completed questionnaires during this time, while the research staff monitored the infant in the same room.

#### 2.2.2 Sample collection, processing, and immunoassay

##### 2.2.2.1 Saliva Sample Collection

Maternal saliva samples were collected using cylindrical cotton swab (Salivette^®^; Sarstedt, Nümbrecht, Germany). The mother placed a swab in her mouth for about 60 sec or until it was soaked throughout. Infant saliva samples were collected using cylindrical cotton stick specifically designed for infants < 6 months of age (SalivaBio^®^ Infant Swab; Salimetrics, State College, PA). The research staff held one end of the swab and placed the other end under the infant’s tongue and/or inside the cheek for a combined total of 90 to 120 sec, or until the majority of the swab was saturated. We aimed to collect 0.6 to 0.8 ml of saliva from the mother and the infant at each timepoint for OT and VP assay.

##### 2.2.2.2 Blood Sample Collection

Maternal blood samples were collected into a pre-chilled EDTA tube using the previously inserted canula. We aimed to collect a minimum of 1.8 to 2 ml of blood from the mother at each timepoint to ensure that a minimum of 0.8 ml of plasma was available for OT and VP assay.

##### 2.2.2.3 Sample Processing and Immunoassay

Upon collection, saliva swabs were immediately stored in capped storage tubes and placed at 4 °C before being centrifuged at 3000rpm, 4 °C for 10 min. The samples were then aliquoted into 1.5 ml cryovials (two aliquots of 0.3-0.4 ml each, one for OT and one for VP), evaporated to dryness in a SpeedVac concentrator at room temperature, and stored at -80 °C until they were shipped in a single batch for immunoassay. For blood samples, the tubes were immediately stored at 4 °C upon collection before being centrifuged at 1300g, 4 °C for 10 min. A total of 0.8 ml plasma was pipetted into a 2.0 ml cryovial (for both OT and VP) and stored at -80 °C until they were shipped in a single batch for immunoassay. OT and VP levels in the saliva and plasma samples were quantified using a highly sensitive and specific radioimmunoassay (RIAgnosis, Sinzing, Germany). Assay sensitivity was in the 0.1-pg range. Intra- and inter-assay variabilities were < 10%, and cross-reactivity to a variety of other peptides was < 0.7%.

### 2.3 Additional maternal and infant characteristics

#### 2.3.1 Maternal postpartum depression and anxiety

To confirm study eligibility criteria, severity of the mother’s PPD symptoms was measured using the EPDS and CESD-R at study entry. The EPDS is a 10-item self-report questionnaire well-validated and widely used to assess PPD (76, 83). Severity of the mother’s anxiety symptoms was measured using the State Trait Anxiety Inventory (STAI). The STAI is a 40-item self-report questionnaire of anxiety, with widely demonstrated validity and applicability to postpartum mothers (84). The STAI measures both trait anxiety (one’s general disposition toward anxiety) and state anxiety (one’s experience of anxiety at a specific time).

#### 2.3.2 Maternal childhood trauma

The mother’s childhood trauma was measured using the Childhood Trauma Questionnaire (CTQ). The CTQ is one of the most widely used and well-validated self-report measures that provide a reliable retrospective assessment of childhood abuse and neglect before the age of 18 (85). The CTQ consists of 28 items and yields five clinical scales: physical abuse, sexual abuse, emotional abuse, emotional neglect, and physical neglect.

#### 2.3.3 Maternal attachment

The mother’s adulthood attachment was measured using the Experiences in Close Relationships Scale-Revised (ECR). The ECR is one of the most commonly used self-report measures of adulthood attachment. It has extensively tested psychometric properties (86–88) and yields attachment anxiety and attachment avoidance subscales. The mother’s specific attachment to her infant in the postpartum was assessed using the Maternal Postnatal Attachment Scale (MPAS) (89). The MPAS is a 19-item self-report questionnaire developed to assess attachment between postpartum mothers and their infants. Higher scores on the MPAS indicate a higher degree of mother-infant bonding, with subscales representing dimensions of Absence of Hostility, Pleasure in Interaction, and Quality of Attachment.

#### 2.3.4 Maternal reflective functioning

The mother’s reflective functioning was measured using the Parental Reflective Functioning Questionnaire (PRFQ) (90). The PRFQ is an 18-item self-report questionnaire measuring parental reflective functioning for parents of children up to 5 years of age. The PRFQ contains three subscales capturing different dimensions of a parent’s relationship to the mental states of their child. Specifically, the Pre-Mentalizing Modes subscale measures the difficulty of parents’ in making accurate assessments about the mental states of their child, the Certainty of Mental States subscale assesses the extent to which parents acknowledge the lack of clarity when trying to understand their child’s mental states, and the Interest and Curiosity in Mental States subscale is a measure of parents’ interest in the mental states of their child (91, 92).

### 2.4 Statistical methods

All statistical analyses and visualizations were performed with the use of SAS^®^ software (Ver. 9.4). For all analyses, *p*-values were left unadjusted due to the exploratory nature of the study. Thus, *p*-values falling below conventional thresholds are not presented to be significant as such and are only demarcated to indicate findings of potential future interest.

The first phase of analysis consisted of evaluating various sociodemographic and clinical characteristics in the sample, and comparisons were made between the PPD and non-PPD groups using Fisher’s Exact tests for categorical data and Mann-Whitney U tests for continuous data. For questionnaire data evaluating maternal and infant characteristics, comparisons were performed via Mann Whitney U tests and variables with *p* <.05 were flagged for subsequent evaluation of potential interactions.

Missing values in the hormone variables (all missing values were in salivary VP values; see **Supplementary Table 1**) were imputed using stochastic regression imputation (SRI) implemented via Proc MI in SAS. Complete Case Analysis (CCA) was also performed for comparison and no changes were reflected in the substantial conclusions drawn from the statistical tests utilized. Following imputation, changes in hormone levels from baseline were calculated for each timepoint and subsequently divided by baseline levels to generate a derived variable representing relative change at each timepoint. These values were then multiplied by 100% to express the changes in terms of % values. Sample-wide trajectory of change in each hormone from baseline was first visualized using median-connected boxplots and Wilcoxon Signed-Rank tests were performed using the relative change in hormone levels, evaluating their potential differences from zero. Differences in trajectory between PPD and non-PPD groups were visualized utilizing the same boxplot method and between-group comparisons in relative hormone changes were made using Mann-Whitney U tests for each timepoint. All plots were generated using complete cases, while tests were performed using imputed data. Effect sizes were measured by *r* = *Z*/sqrt(*N*). Given the exploratory nature of this study and the limited sample size, we report analyses from the Wilcoxon Signed-Rank and Mann-Whitney U tests as our primary results (93). Additionally, we employ a nonparametric repeated-measures model approach based on rank-transformed data (94), with corresponding statistical methods and results presented in the **Supplementary Material**.

Correlations between sample-wide changes for maternal and infant salivary measurements were evaluated for both OT and VP using complete cases. Differences between these correlations across PPD groups were visualized by fitting regression lines to each group separately.

**Figure 2:**
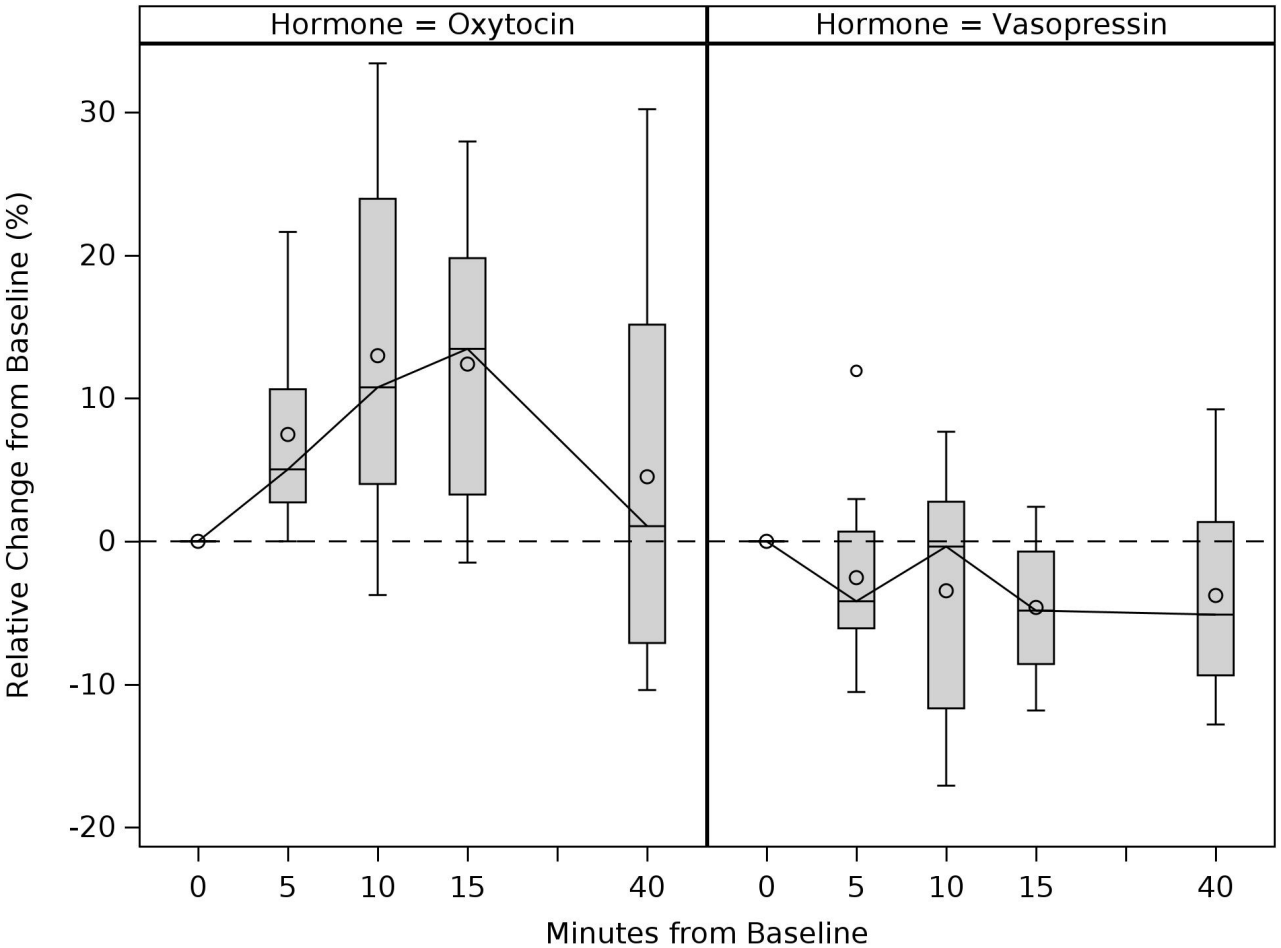
Sample-wide trajectories of change in the mother’s plasma oxytocin and vasopressin levels before, during, and after mother-infant interaction. Boxes connected by median for each time period and represent complete cases. Time period 0min refers to pre-interaction baseline. Mother-infant interaction took place between 0 to 15min and 40min represents post-interaction levels (i.e., 25min after the mother-infant interaction concluded).

The final phase of analysis consisted of exploring inter-relationships among PPD, hormone level changes, and maternal and infant characteristics of interest. Maternal PPD was evaluated both as a binary (PPD vs. non-PPD) and continuous variable (EPDS scores). For hormone levels, we evaluated relative change in maternal plasma OT at 15-min, given that this timepoint represented the greatest change from baseline in the full sample (see **Figure 2**). For maternal and infant characteristics, we limited our evaluation to the variables that were observed to have differential patterns in PPD vs. non-PPD groups. These variables included: breastfeeding status/duration, CTQ Emotional Abuse, ECR Attachment Anxiety, all MPAS subscales, and PRFQ Certainty about Mental States/Pre-Mentalizing Modes subscales (we excluded psychiatric variables [e.g., depression, anxiety, treatment history] that were, by design, expected to be different in PPD vs. non-PPD groups given the eligibility criteria). To visualize potential interactions, scatterplots were created with data being classified by an appropriate grouping variable. Prior to plotting, all variables were standardized to allow comparison on the same unit-scale. Scores for ECR Attachment Anxiety and CTQ Emotional Abuse were grouped on the basis of being either above or below the median, while breastfeeding status/duration was grouped as being either at or below the median, with the median breastfeeding category representing “currently breastfeeding,” which included the majority (*n* = 7) of mothers in the sample (*n* = 12). Categories below the median were breastfeeding durations of 4 to 6 months, 7 weeks to 3 months, 3 to 6 weeks, and 2 weeks or less, meaning that these categories could effectively be interpreted as “not currently breastfeeding.” In all plots, regression lines were fit to each category/group separately and potential interactions were identified through visual inspection of differences in slope between groups.

Given the field’s longstanding interest in the relationship between breastfeeding and OT functions, we additionally report spearman rank correlations between breastfeeding duration and maternal OT levels, stratified by PPD groups, in the **Supplementary Material**.

## 3 Results

### 3.1 Description of sample

Sociodemographic and clinical characteristics of the sample are presented in **Table 1**. All our enrolled mothers were White, non-Hispanic, married, and living with spouse, with the majority of the mothers having a college or graduate degree. Differences were found between groups for breastfeeding status (*p* = .046), prior history of outpatient psychiatric care (*p* = .01), and prior history of psychiatric medication treatment (*p* = .01). More mothers in the PPD group had a history of outpatient psychiatric care and psychiatric medication treatment, and reported that they were no longer breastfeeding at the time of the study. A detailed list of medications taken by mothers at the time of the study is provided in **Supplementary Table 2**.

**Table 1:** Maternal and infant characteristics of PPD vs. Non-PPD groups.

	Non-PPD (*n*=7)	PPD (*n*=5)	Total (*N*=12)	*p*-value
Age, M (*SD)*				
Maternal Age	31.29 (4.86)	31.20 (3.11)	31.25 (4.05)	0.935
Infant Age	13.78 (3.49)	13.86 (7.20)	13.81 (5.05)	0.745
Infant Sex, *n* (%)				
Male	3	1	4 (33.33)	0.576
Female	4	4	8 (66.67)	
Maternal Parity, *n* (%)				
Primiparous	1	3	4 (33.33)	0.222
Multiparous	6	2	8 (66.67)	
Maternal Education, *n* (%)				
High School Graduate	0	1	1 (8.33)	0.735
College or University Graduate	2	1	3 (25)	
Graduate Professional Training	5	3	8 (66.67)	
Annual Family Income, *n* (%)				
$30,001 – 45,000	0	1	1 (8.33)	0.5
$45,001 – 70,000	3	1	4 (33.33)	
$70,000 – 100,00	1	2	3 (25)	
Above $100,000	3	1	4 (33.33)	
Maternal Work From Home Status, *n* (%)				
Yes	4	1	5 (41.67)	0.293
No	3	4	7 (58.33)	
Mode of Delivery, *n* (%)				
Vaginal	6	4	10 (83.33)	1.00
Cesarean Section	1	1	2 (16.67)	
Breastfeeding Status, *n* (%)				
Currently Breastfeeding	6	1	7 (58.33)	0. 046*
No Longer Breastfeeding				
Breastfed 4–6 Months	0	1	1 (8.33)	
Breastfed 7 Weeks - 3 Months	0	1	1 (8.33)	
Breastfed 3–6 Weeks	0	1	1 (8.33)	
Breastfed 2 Weeks or Less	1	1	2 (16.67)	
Maternal History of Outpatient Psychiatric Treatment, *n* (%)				
Yes	0	4	4 (33.33)	0.01**
No	7	1	8 (66.67)	
Maternal History of Psychiatric Medication Treatment, *n* (%)				
Yes	0	4	4 (33.33)	0.01**
No	7	1	8 (66.67)	
Maternal History of Psychotherapy Treatment, *n* (%)				
Yes	1	3	4 (33.33)	0.222
No	6	2	8 (66.67)	

Between-group comparisons were made using Fisher’s Exact test for categorical data and Mann-Whitney U tests for continuous data.

^*^p <.05. ^**^p <.01.

Between-group differences in the questionnaire data are presented in **Table 2**. As expected given our eligibility criteria, results of the Mann-Whitney U test confirmed higher depression scores in the PPD compared to the non-PPD group, as reflected in the EPDS (*Z* = 2.780, *p* = .005, *r* = .803) and CESD-R (*Z* = 2.785, *p* = .005, *r* = .804) scores. Furthermore, compared to mothers in the non-PPD group, mothers in the PPD group scored higher in STAI Trait Anxiety (*Z* = 2.766, *p* = .006, *r* = .695), STAI State Anxiety (*Z* = 2.775, *p* = .006, *r* = .700), CTQ Emotional Abuse (*Z* = 2.531, *p* = .011, *r* = .731), ECR Attachment Anxiety (Z = 2.766, *p* = .006, *r* = .798), and PRFQ Pre-Mentalizing Modes (*Z* = 2.475, *p* = .013, *r = .*715), while scoring lower for MPAS Absence of Hostility (*Z* = -2.453, *p* = .014, *r* = -.708), MPAS Pleasure in Interaction (*Z* = -2.367, *p* = .018, *r* = -.683), MPAS Quality of Attachment (*Z* = -2.785, *p* = .005, *r* = -.804), and PRFQ Certainty about Mental States (*Z* = -2.286, *p* = .022, *r* = -.660).

**Table 2:** Mann-Whitney U test for PPD vs. Non-PPD group differences in questionnaire data.

Variable	Non-PPD (*n*=7)		PPD (*n*=5)		*Z*	*r*	*p*-value
	Median	Mean rank	Median	Mean rank			
Maternal Depression							
EPDS	1.000	4.000	18.000	10.000	2.780	0.803	0.005**
CESD-R	1.000	4.000	39.000	10.000	2.785	0.804	0.005**
Maternal Anxiety							
STAI Trait Anxiety	25.000	4.000	56.000	10.000	2.766	0.695	0.006**
STAI State Anxiety	21.000	4.000	52.000	10.000	2.775	0.700	0.006**
Maternal Trauma							
CTQ Emotional Abuse	5.000	4.286	11.000	9.600	2.531	0.731	0.011*
CTQ Emotional Neglect	6.000	5.857	8.000	7.400	0.674	0.194	0.501
CTQ Physical Abuse	5.000	5.643	6.000	7.700	0.969	0.280	0.333
CTQ Physical Neglect	5.000	5.786	6.000	7.500	0.817	0.236	0.414
CTQ Sexual Abuse	5.000	6.000	5.000	7.200	1.014	0.293	0.311
Maternal Adulthood Attachment							
ECR Attachment Anxiety	1.333	4.000	3.111	10.000	2.766	0.798	0.006**
ECR Attachment Avoidance	1.500	5.714	2.222	7.600	0.813	0.235	0.416
Maternal Postnatal Attachment to Infant							
MPAS Absence of Hostility	22.600	8.714	11.000	3.400	-2.453	-0.708	0.014*
MPAS Pleasure in Interaction	22.000	8.643	16.000	3.500	-2.367	-0.683	0.018*
MPAS Quality of Attachment	43.600	9.000	30.300	3.000	-2.785	-0.804	0.005**
Maternal Reflective Functioning							
PRFQ Certainty about Mental States	4.833	8.571	2.500	3.600	-2.286	-0.660	0.022*
PRFQ Interest and Curiosity in Mental States	5.333	5.214	6.333	8.300	1.393	0.402	0.164
PRFQ Pre-Mentalizing Modes	1.167	4.286	2.833	9.600	2.475	0.715	0.013*

All between-group comparisons were made using Mann-Whitney U tests. *r*-statistic is reported as a measure of effect size; *r* = *Z*/sqrt(*N*). EPDS, Edinburgh Postnatal Depression Scale; CESD-R, Center for Epidemiologic Studies Depression Scale Revised; STAI, The State-Trait Anxiety Inventory; CTQ, Childhood Trauma Questionnaire; ECR, Experiences in Close Relationships-Revised; MPAS, Maternal Postnatal Attachment Scale; PRFQ, Parental Reflective Functioning Questionnaire.

^*^p <.05. ^**^p <.01.

### 3.2 Sample-wide trajectories of OT and VP

**Figure 3:**
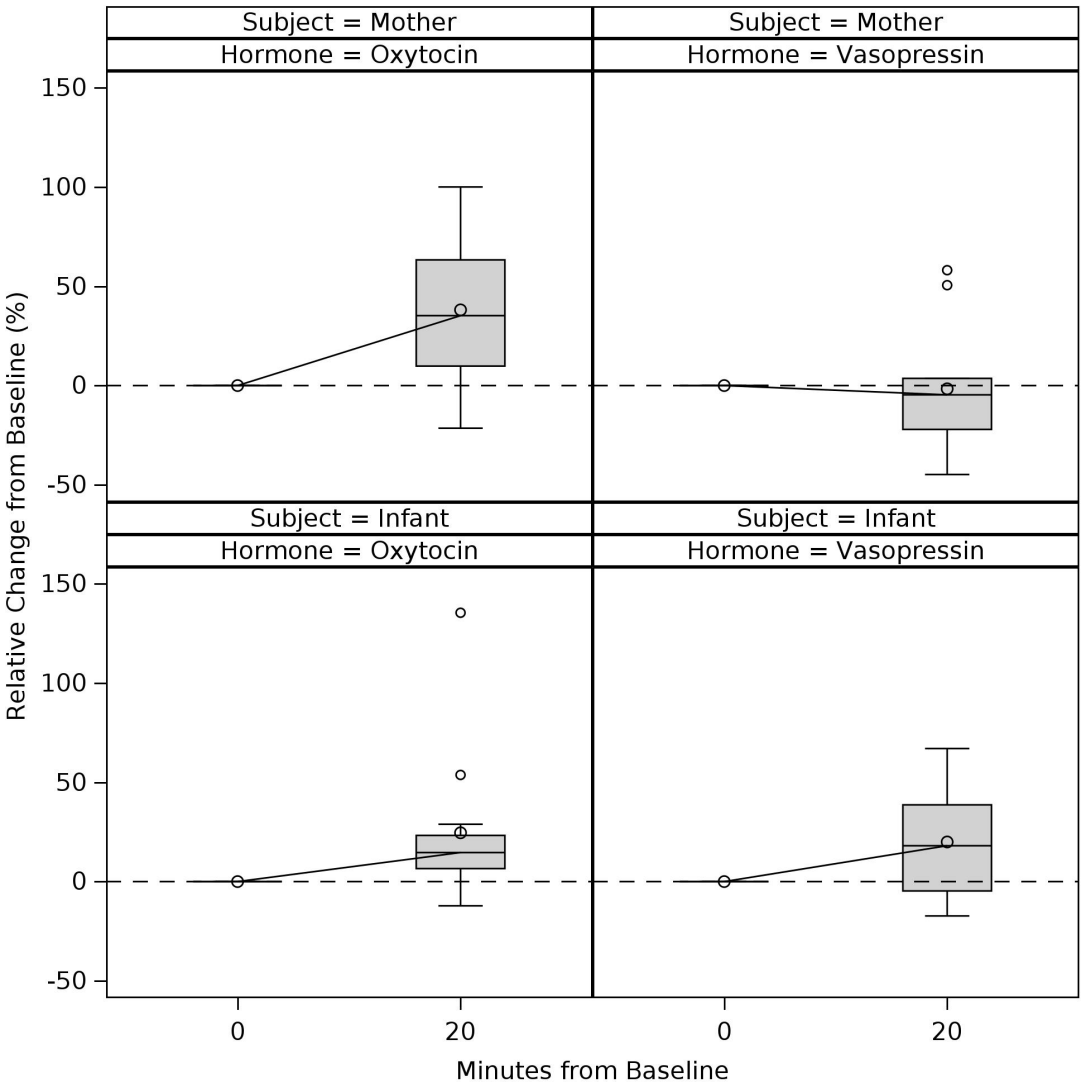
Sample-wide trajectories of change in the mother’s and infant’s salivary oxytocin and vasopressin levels before and after mother-infant interaction. Boxes connected by median for each time period and represent complete cases. Time-period 0min refers to pre-interaction baseline. Mother-infant interaction took place between 0 to 15min and post-interaction saliva was collected at 20min (i.e., 5min after the mother-infant interaction concluded).

Sample-wide descriptive statistics of OT and VP measurements at each timepoint are presented in **Table 3**. Boxplots shown in **Figures 2** and **3** illustrate sample-wide trajectories of OT and VP responses before, during, and after the mother-infant interaction. **Figure 2** shows maternal plasma measurements and **Figure 3** shows maternal and infant salivary measurements. Median plasma OT levels appeared to gradually increase in the first 15 minutes of the mother-infant interaction (between 0 to 15 minutes) and gradually decrease toward its baseline level between 15- to 40-minutes. Although there appeared to be a slight decrease in plasma VP during and after the mother-infant interaction, median VP levels overall exhibited minimal changes throughout the interaction. For salivary measurements, there were median increases in both the mother and infant OT 20 minutes from baseline, in addition to an increase in infant VP. Median maternal salivary VP exhibited minimal change. **Table 4** presents a sample-wide examination (across both PPD and non-PPD groups) of % change values in OT and VP measurements during and after the mother-infant interaction. For OT measurements, increases were observed in maternal plasma at 5 min (median = 5.03, *Z* = 2.589, *p* = .001, *r* = .747), 10 min (median = 10.77, *Z* = 2.746, *p* = .003, *r* = .793), and 15 min (median = 13.47, *Z* = 2.903, *p* = .002, *r* = .838), and in maternal (median = 35.21, *Z* = 2.667, *p* = .005, *r* = .770) and infant saliva (median = 14.63, *Z* = 2.667, *p* = .005, *r* = .770) at 20 min following the start of the interaction. For VP measurements, a decrease was found in maternal plasma at 15 min (median = -4.85, *Z* = -2.510, *p* = .009, *r* = -.725) following the start of the interaction.

**Table 3:** Descriptive statistics of OT and VP levels: sample-wide and PPD subgroups.

Variable	*M (SD)*		
	Non-PPD (*n*=7)	PPD (*n*=5)	Total (*N*=12)
Oxytocin (OT)			
Maternal Plasma			
Baseline Plasma (OT1p)	3.18 (0.14)	3.26 (0.17)	3.21 (0.15)
5-min Plasma (OT2p)	3.48 (0.35)	3.42 (0.24)	3.45 (0.30)
10-min Plasma (OT3p)	3.65 (0.49)	3.60 (0.31)	3.63 (0.41)
15-min Plasma (OT4p)	3.61 (0.39)	3.62 (0.39)	3.61 (0.37)
40-min Plasma (OT6p)	3.16 (0.27)	3.63 (0.58)	3.36 (0.47)
Maternal Saliva			
Baseline Salivary (OT1s)	1.01 (0.17)	1.42 (0.41)	1.18 (0.35)
20-min Salivary (OT5s)	1.46 (0.19)	1.64 (0.22)	1.53 (0.21)
Infant Saliva			
Baseline Salivary (OT1s)	1.19 (0.25)	1.29 (0.17)	1.23 (0.22)
20-min Salivary (OT5s)	1.47 (0.30)	1.50 (0.28)	1.49 (0.28)
Vasopressin (VP)			
Maternal Plasma			
Baseline Plasma (VP1p)	3.27 (0.21)	3.46 (0.05)	3.35 (0.18)
5-min Plasma (VP2p)	3.23 (0.23)	3.30 (0.14)	3.26 (0.19)
10-min Plasma (VP3p)	3.14 (0.21)	3.34 (0.27)	3.22 (0.25)
15-min Plasma (VP4p)	3.13 (0.22)	3.28 (0.21)	3.19 (0.22)
40-min Plasma (VP6p)	3.21 (0.17)	3.21 (0.08)	3.21 (0.13)
Maternal Saliva			
Baseline Salivary (VP1s)	1.34 (0.24)	1.42 (0.33)	1.37 (0.26)
20-min Salivary (VP5s)	1.19 (0.21)	1.52 (0.35)	1.29 (0.28)
Infant Saliva			
Baseline Salivary (VP1s)	1.27 (0.18)	1.19 (0.20)	1.23 (0.18)
20-min Salivary (VP5s)	1.42 (0.29)	1.54 (0.48)	1.47 (0.34)

All units are pg/ml. See **Figure 1** for details on timepoints for OT and VP sample collection. There were missing values for VP salivary data (see **Supplementary Table 1** for details).

**Table 4:** Wilcoxon signed-rank test for sample-wide changes in OT and VP levels from baseline.

Variable	Median	*Z*	*r*	*p*-value
Oxytocin (OT)				
Maternal Plasma				
5-min Plasma Oxytocin % Change	5.03	2.589	0.747	0.001***
10-min Plasma Oxytocin % Change	10.77	2.746	0.793	0.003**
15-min Plasma Oxytocin % Change	13.47	2.903	0.838	0.002**
40-min Plasma Oxytocin % Change	1.06	0.784	0.226	0.470
Maternal Saliva				
20-min Salivary Oxytocin % Change	35.21	2.667	0.770	0.005**
Infant Saliva				
20-min Salivary Oxytocin % Change	14.63	2.667	0.770	0.005**
Vasopressin (VP)				
Maternal Plasma				
5-min Plasma Vasopressin % Change	-4.19	-1.647	-0.476	0.110
10-min Plasma Vasopressin % Change	-0.365	-1.020	-0.294	0.339
15-min Plasma Vasopressin % Change	-4.85	-2.510	-0.725	0.009**
40-min Plasma Vasopressin % Change	-5.13	-1.647	-0.476	0.110
Maternal Saliva				
20-min Salivary Vasopressin % Change	-4.66	-0.628	-0.181	0.569
Infant Saliva				
20-min Salivary Vasopressin % Change	-1.48	0.314	0.091	0.791

Values represent relative change in OT and VP levels from pre-interaction baseline (i.e., 0min). Change scores between each timepoint and baseline were calculated and divided by the baseline value, before being converted to % changes by multiplying by 100%. *r*-statistic is reported as a measure of effect size; *r* = *Z*/sqrt(*N*).

^*^p <.05. ^**^p <.01. ^***^p <.001.

### 3.3 Comparison of OT and VP trajectories between PPD and non-PPD groups

**Figure 4:**
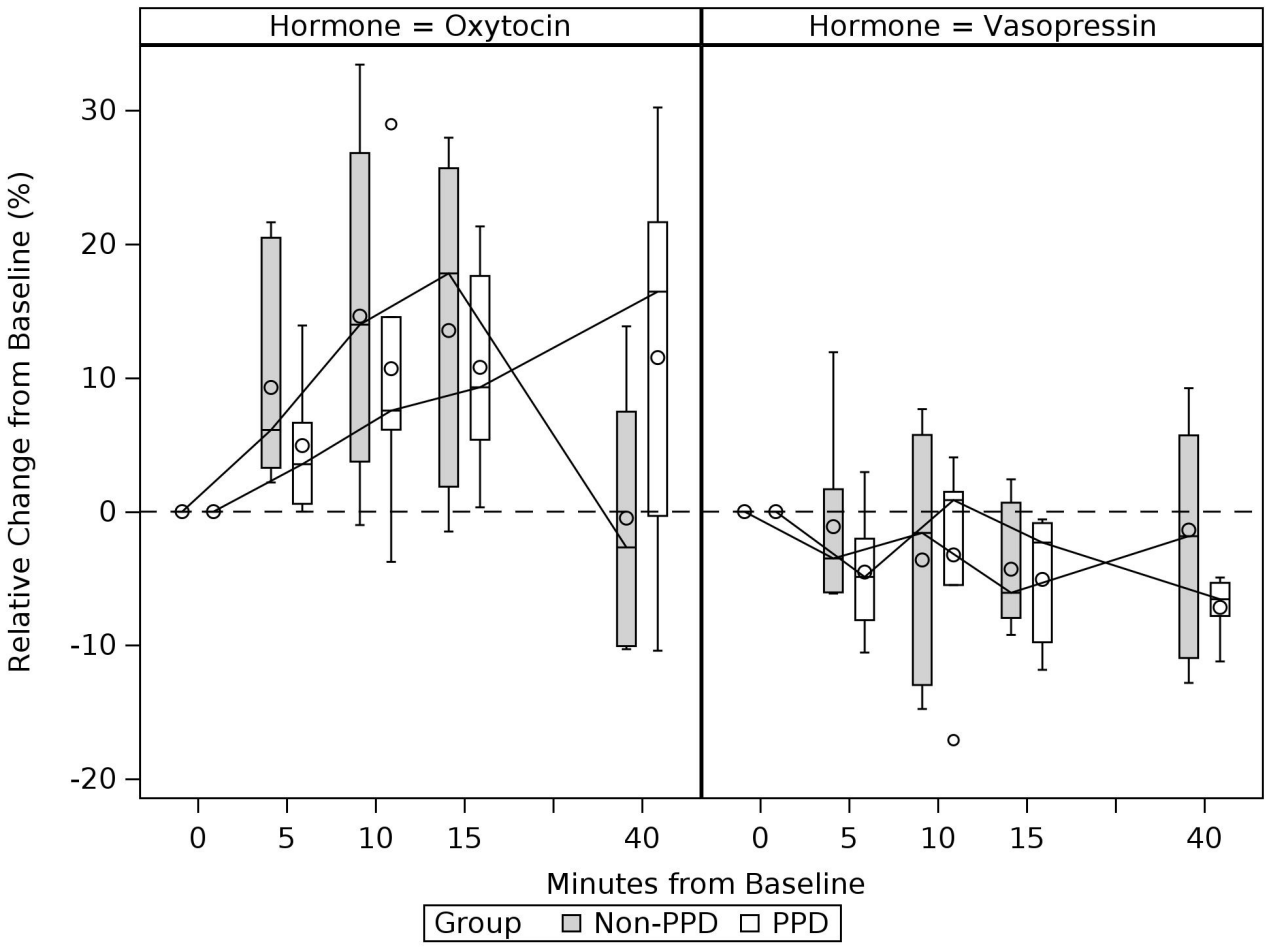
Trajectories of change in the mother’s plasma oxytocin and vasopressin levels during and after mother-infant interaction: PPD vs. Non-PPD Groups. Boxes connected by median for each time-period and grouped according to PPD status. Values represent complete cases. Time-period 0min refers to pre-interaction baseline and subsequent timepoints refer to the number of minutes elapsed since the start of the mother-infant interaction. Mother-infant interaction took place between 0 to 15min.

**Figure 5:**
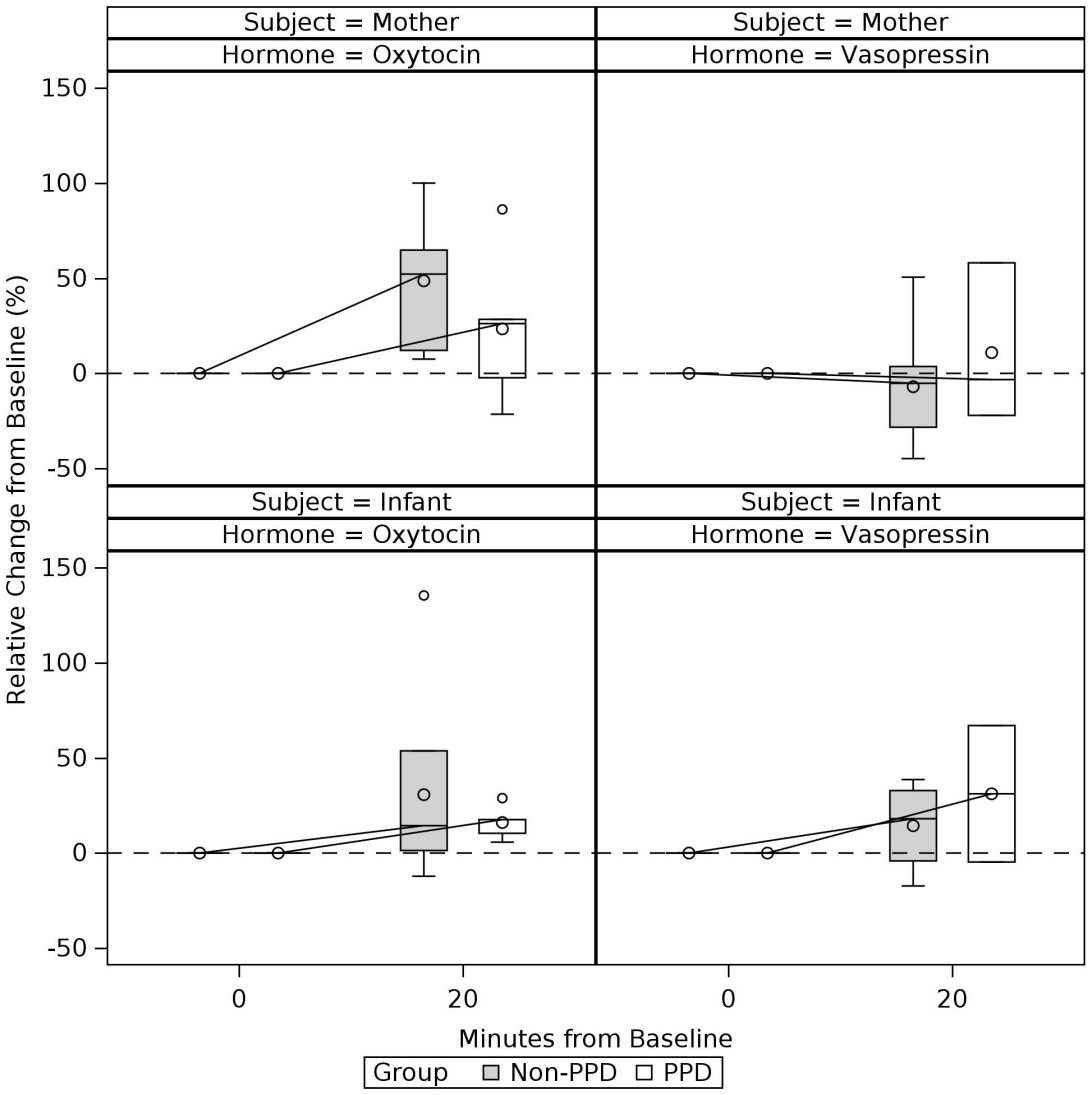
Trajectories of change in the mother’s and infant’s salivary oxytocin and vasopressin levels during and after mother-infant interaction: PPD vs. Non-PPD Groups. Boxes connected by median for each time period and grouped according to PPD status. Values represent complete cases. Time-period 0min refers to pre-interaction baseline. Mother-infant interaction took place between 0 to 15min and post-interaction saliva was collected at 20min (i.e., 5min after the mother-infant interaction concluded).

Descriptive statistics of OT and VP measurements at each timepoint are presented in **Table 3** for PPD and non-PPD groups. Grouped boxplots in **Figures 4** and **5** illustrate potential differences in OT and VP trajectories between the PPD and non-PPD groups. **Figure 4** shows maternal plasma measurements and **Figure 5** shows maternal and infant salivary measurements. For plasma measurements, median OT levels in the non-PPD group showed an expected increase from pre-interaction baseline, achieving its peak levels at around 15 min following the start of the mother-infant interaction. This was then followed by a decline, reaching its pre-interaction baseline at 40 min. The PPD group appeared to show a similar, albeit more gradual/less steep, upward trend in median OT levels between pre-interaction baseline and 15 min. However, unlike the non-PPD group whose OT levels decreased following the conclusion of the mother-infant interaction, the PPD group continued to display sustained elevated levels of OT even at the 40 min time point. Differences in plasma VP response were less clear, although both groups showed a general decrease in median plasma VP levels. A slight increase in median plasma VP towards baseline seemed to be indicated from 15 to 40-minutes in the non-PPD group.

Although maternal salivary OT showed a median increase from pre-interaction baseline to 20 min for both groups, OT showed a slightly steeper increase in the non-PPD group, consistent with the pattern seen for plasma OT. Median increases in infant salivary OT were also observed for both groups, however infants of mothers in the non-PPD group displayed greater upward spread in OT levels compared to infants of mothers in the PPD group. Maternal salivary VP measurements showed minimal change in both groups, while infant salivary VP measurements showed median increases for both groups.

As expected, given the small sample size, no between-group differences (PPD vs. non-PPD group) in OT or VP levels survived statistical threshold of *p* <.05 when using Mann-Whitney U tests (**Supplementary Table 3**). Results from the non-parametric repeated measures models are presented in **Supplementary Table 4** and generally align with findings from the Wilcoxon Signed-Rank and Mann-Whitney U tests, reported in **Table 4** and **Supplementary Table 3**, respectively.

### 3.4 Associations between changes in the mother’s and infant’s salivary OT and VP levels

**Figure 6:**
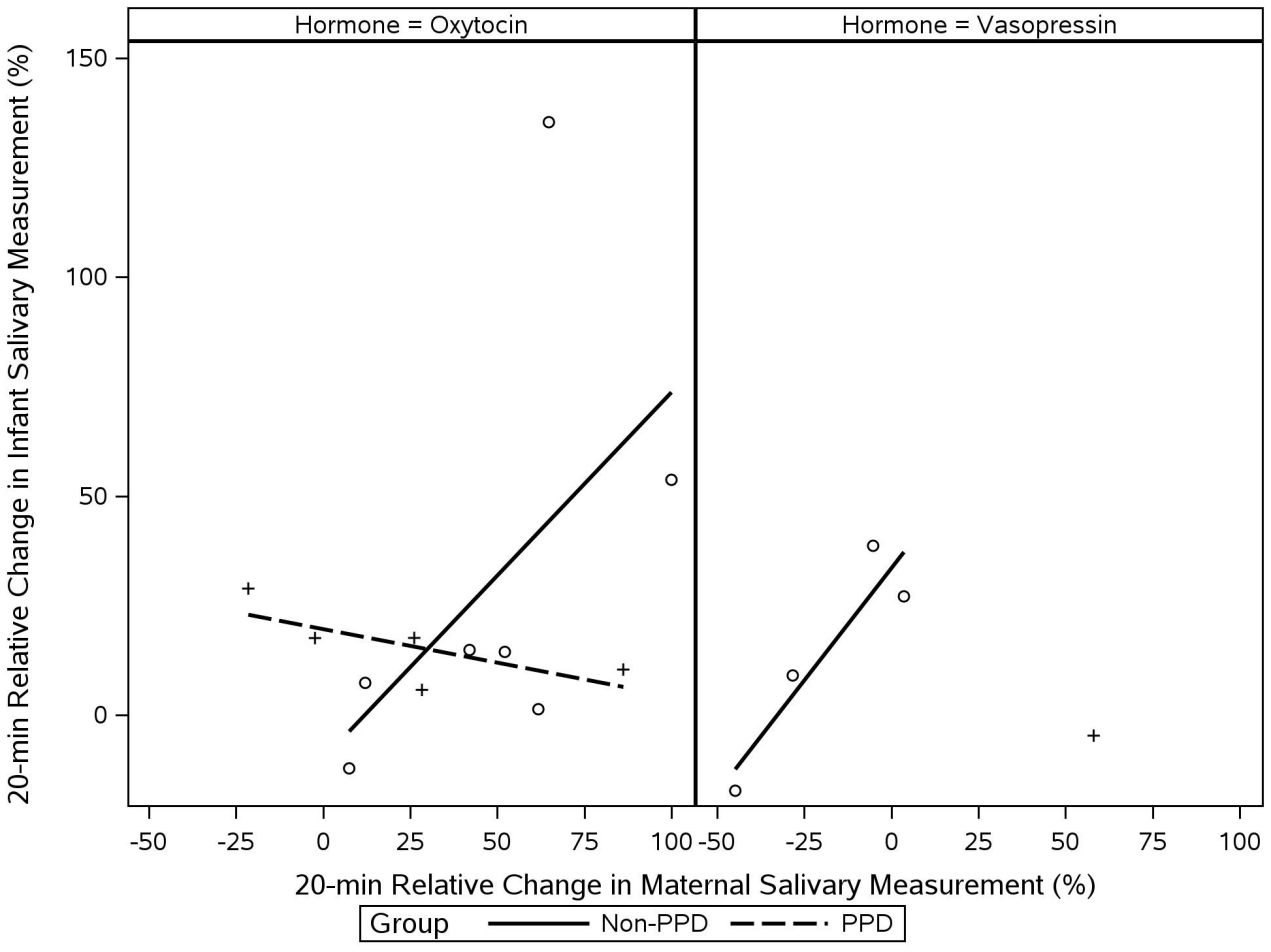
Associations between changes in the mother’s and infant’s salivary oxytocin and vasopressin levels. Values represent complete cases. Given the missing values in salivary VP measurements, only one complete mother-infant pair was available for VP measurements in the PPD group. Therefore, this relationship could not be visualized for the PPD group.

Across the sample, we observed positive associations between relative changes in the mother’s and infant’s hormone levels (**Table 5**). Such intergenerational association was stronger for maternal and infant OT changes (*r* = 0.34) than for maternal and infant VP changes (*r* = 0.09). The associations between OT and VP changes were generally negative across the sample. This was true both within and between maternal and infant measurements. When stratifying by PPD and non-PPD groups (**Figure 6**), the visualization revealed that maternal and infant OT changes were positively correlated in the non-PPD group but showed a slight negative trend in the PPD group. Maternal and infant VP changes were also positively correlated in the non-PPD group; however, this relationship could not be visualized in the PPD group due to the presence of only a single complete measurement pair.

**Table 5:** Descriptive statistics and correlations for changes in the mother’s and the infant’s salivary oxytocin and vasopressin levels.

Variable	*n*	*M*	*SD*	1	2	3	4
1. Maternal Oxytocin % Change	12	38.11	36.51	–			
2. Maternal Vasopressin % Change	10	-1.64	32.83	-0.11	–		
3. Infant Oxytocin % Change	12	24.58	38.36	0.34	-0.32	–	
4. Infant Vasopressin % Change	6	19.96	30.75	-0.35	0.09	-0.49	–

Values represent complete cases. Values indicate % change values from pre-interaction baseline (i.e., 0min) to 20-min saliva measurement.

### 3.5 Inter-relationships among maternal PPD, maternal OT, and maternal/infant characteristics

**Figure 7:**
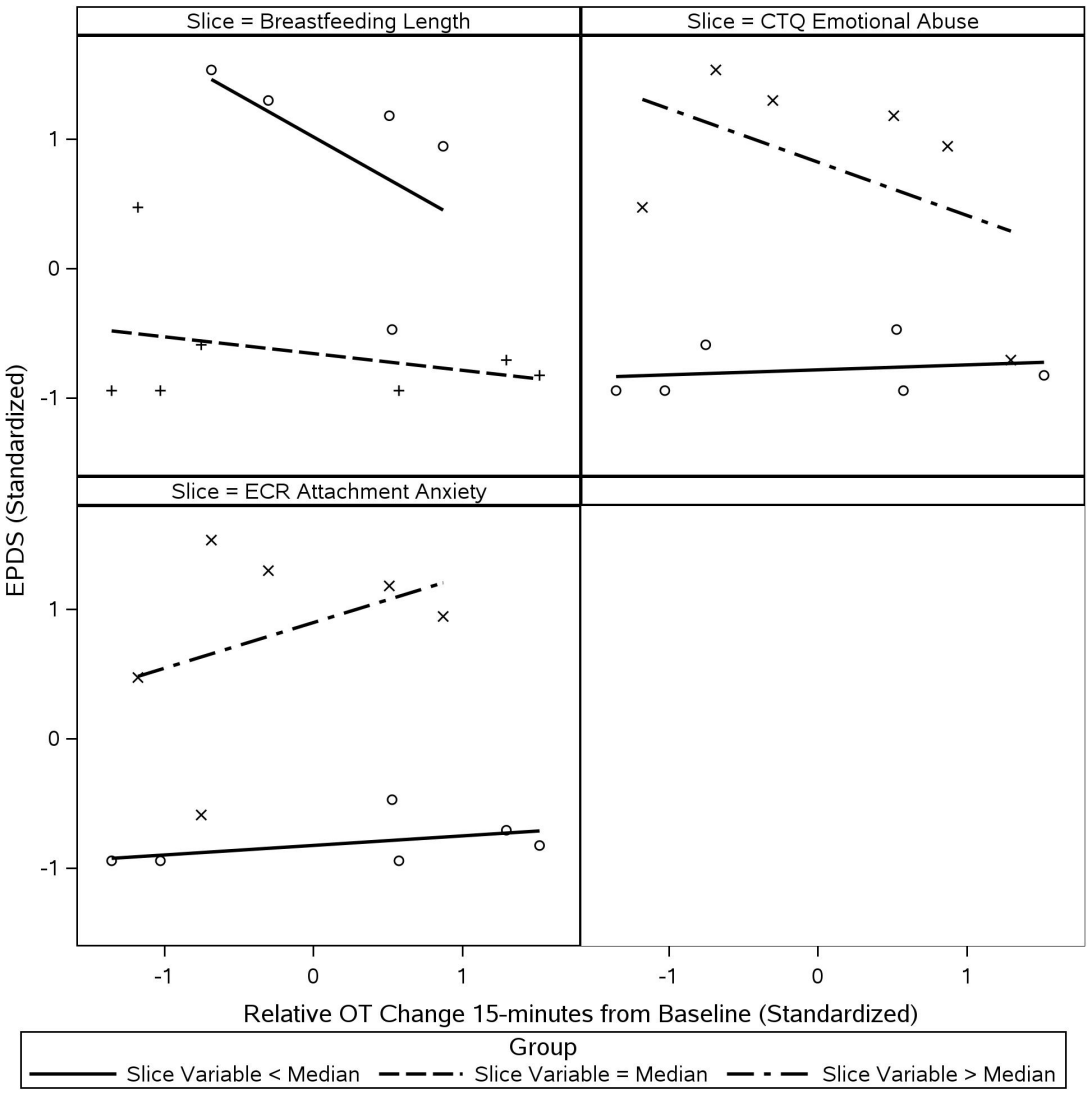
Inter-relationships among maternal PPD symptoms, maternal OT, and several maternal/infant characteristics. Scatterplots illustrate EPDS scores predicted by changes in maternal OT at 15-min from baseline, with data sliced by breastfeeding status/duration, CTQ emotional abuse, and ECR attachment anxiety. Data was grouped based on position relative to the median of the chosen slice variable. All values were standardized prior to plotting and regression lines were fit to each group separately to evaluate visual differences in slope. Median breastfeeding category was “currently breastfeeding,” which represented the majority (*n*=7) of mothers in the sample (*n*=12). Categories below the median were 4–6 months, 7 weeks-3 months, 3–6 weeks, and < 2 weeks. Grouping for breastfeeding length can effectively be interpreted as “= Median”: “currently breastfeeding” and “< Median”: “not currently breastfeeding.” Median ECR Attachment Anxiety was 1.94 and median CTQ Emotional Abuse was 6.5. Means and SDs were as follows: EPDS: 8.00 ± 8.46 and 15-min OT Change: 12.40 ± 10.26. EPDS, Edinburgh Postnatal Depression Scale; CTQ, Childhood Trauma Questionnaire; ECR, Experiences in Close Relationships Scale-Revised.

**Figure 7** illustrates how the associations between maternal PPD severity (EPDS) and maternal OT response (15-min from baseline) varied as a function of maternal breastfeeding status/duration, maternal history of emotional abuse (CTQ), and maternal adulthood attachment anxiety (ECR). In mothers who were no longer breastfeeding, maternal OT response and PPD severity appeared to show a negative relationship, while such a relationship was not apparent in those who were still breastfeeding. Maternal OT response and PPD severity also appeared to show a negative association in mothers with higher levels of emotional abuse relative to the sample, while showing a positive association in mothers with higher levels of attachment anxiety relative to the sample. Such associations were not apparent in mothers with lower histories of emotional abuse and mothers with lower attachment anxiety.

**Figure 8:**
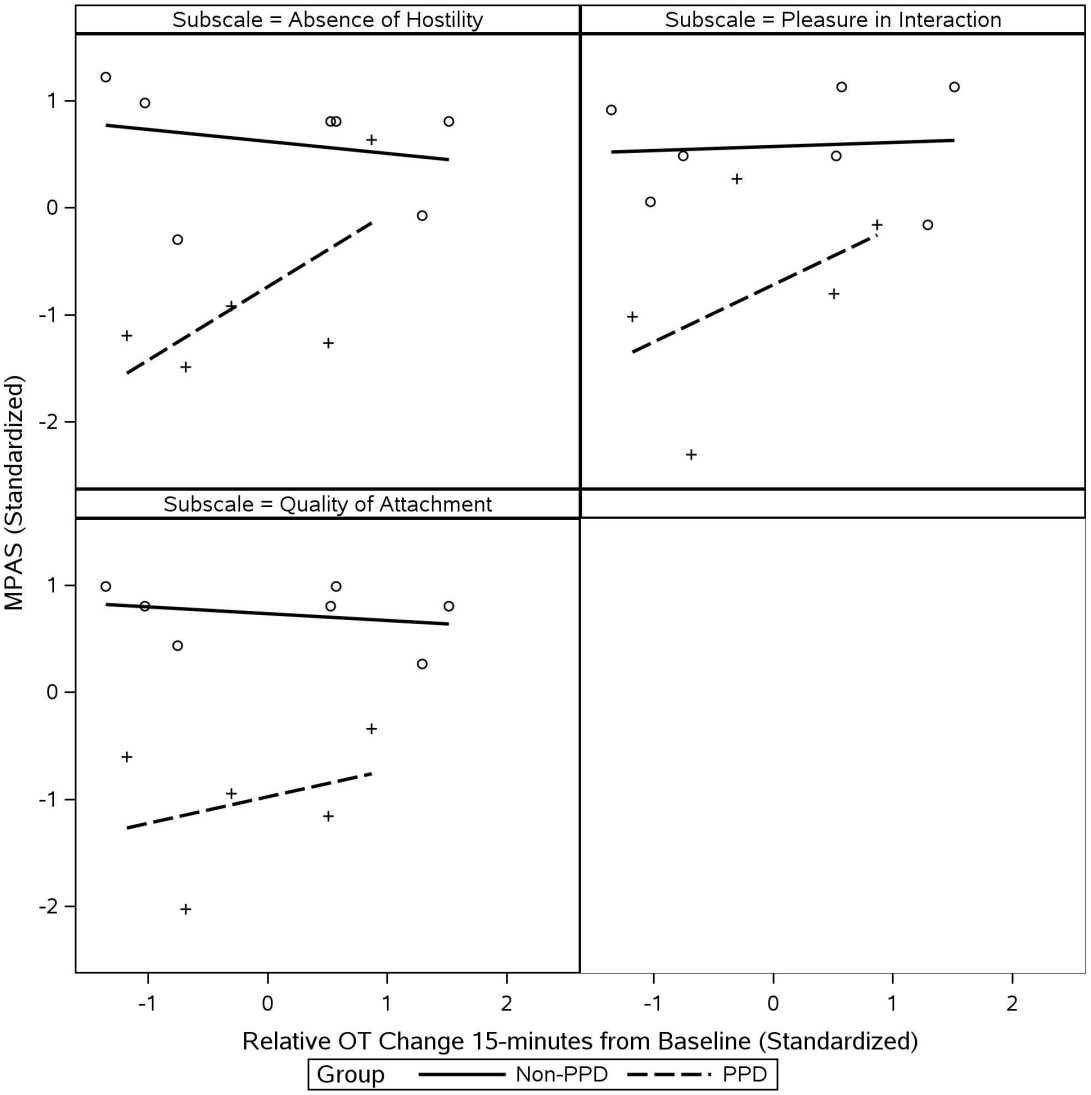
Inter-relationships among maternal PPD status, maternal OT, and maternal postnatal attachment to infant. Scatterplots illustrate each of the MPAS subscales predicted by changes in maternal OT at 15-min from baseline, with data sliced by PPD status. *Y*-axis values represent score of each MPAS subscale. All values were standardized prior to plotting and regression lines were fit to each group separately to evaluate visual differences in slope. Means and SDs were as follows: MPAS Absence of Hostility: 17.93 ± 5.79; MPAS Pleasure in Interaction: 19.75 ± 4.65; MPAS Quality of Attachment: 37.50 ± 7.58; and 15-min OT Change: 12.40 ± 10.26. MPAS, Maternal Postnatal Attachment Scale.

**Figure 8** illustrates how maternal PPD status (PPD vs. non-PPD groups) interacted with maternal OT response (15-min from baseline) to predict maternal postnatal attachment to infant (MPAS subscales). Mothers in the non-PPD group generally reported higher levels of postnatal attachment to their infants (all MPAS subscales [Absence of Hostility, Pleasure in Interaction, and Quality of Attachment]) than mothers in the PPD group. However, for mothers in the PPD group, their attachment to their infants (all MPAS subscales) was positively associated with the magnitude of their OT increase while interacting with their infants. There appeared to be little evidence of such relationship for mothers in the non-PPD group.

**Figure 9:**
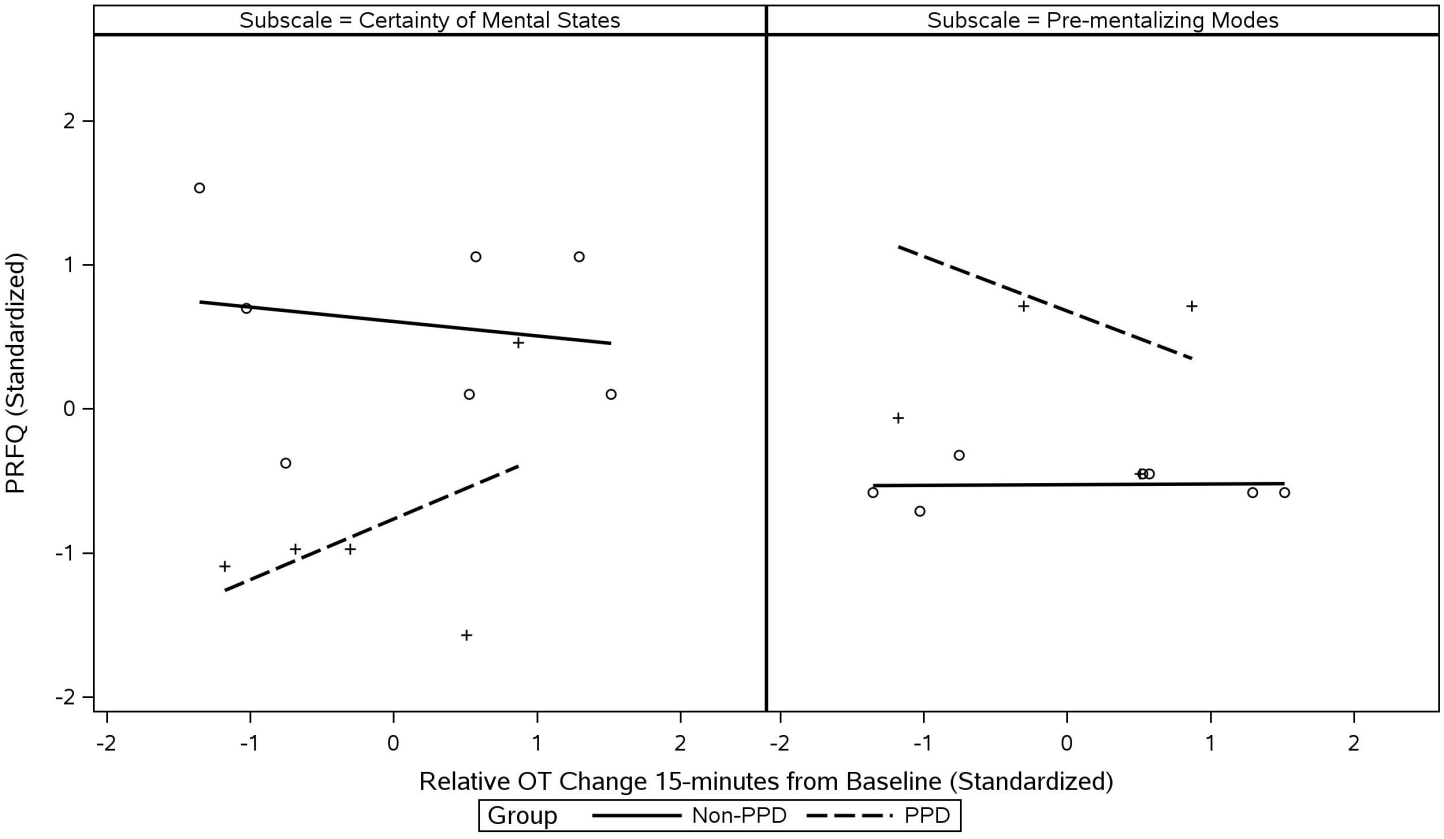
Inter-relationships among maternal PPD, maternal OT, and maternal reflective functioning. Scatterplots illustrate two of the PRFQ subscales predicted by changes in maternal OT at 15-min from baseline, with data sliced by PPD status. *Y*-axis values represent score of each PRFQ subscale. All values were standardized prior to plotting and regression lines were fit to each group separately to evaluate visual differences in slope. Means and SDs were as follows: PRFQ Certainty of Mental States: 3.86 ± 1.40; PRFQ Pre-Mentalizing Modes: 1.92 ± 1.29; and 15-min OT Change: 12.40 ± 10.26. PRFQ, Parental Reflective Functioning Questionnaire.

**Figure 9** similarly illustrates how PPD status (PPD vs. non-PPD groups) interacted with maternal OT response (15-min from baseline) when predicting the Certainty of Mental States and Pre-Mentalizing Modes subscales of the PRFQ. Mothers in the PPD group generally reported lower levels of Certainty of Mental States and higher levels of Pre-Mentalizing Modes than mothers in the non-PPD group. However, for mothers in the PPD group, the magnitude of their OT increase during interaction with their infants appeared to show positive associations with the Certainty of Mental States subscale and negative associations with the Pre-Mentalizing Modes subscale scores. We observed little to no such association in the non-PPD group.

## 4 Discussion

### 4.1 Sample-wide temporal trajectories of OT and VP

The present study is one of the first to perform serial measurements of OT and VP in both the mother and the infant before, during, and after mother-infant interaction to provide descriptive data systematically tracking changes in OT and VP in response to the interaction. Across our sample, we observed increases in maternal OT levels at 5-, 10-, 15- (plasma), and 20- (saliva) minutes following the onset of the interaction, before OT gradually decreased and reached its pre-interaction baseline level at 40 min following the onset of the interaction (i.e., 25 min following the conclusion of the interaction). The trajectory of maternal VP was less clear but we observed a decrease in maternal VP at 15-minute (plasma) following the onset of the interaction. Across our sample, infants showed an increase in both OT and VP (saliva) at 20 min following the onset of the interaction. Despite a rapidly growing interest in measuring peripheral OT levels as a potential marker of socioemotional functions, the field has continued to lack a fundamental understanding of when and how OT/VP levels are expected to rise during social interactions, when and how they are expected to return to their baseline levels, and whether and how these patterns may vary across different types of peripheral samples. By systematically tracking temporal trajectories of plasma and salivary OT/VP changes in mothers and infants following a social paradigm, the present study provides descriptive data illustrating expected trajectories of the two hormones.

### 4.2 Temporal trajectories of OT and VP: PPD vs. non-PPD mothers

To our knowledge, the present study represents the first attempt to illustrate potential differences in these temporal trajectories of OT and VP changes in mothers with and without PPD. We observed that mothers without PPD displayed OT trajectories that were characterized by an increase in OT levels during interactions with their infants (0 to 15 minutes in the present study) followed by a return to its pre-interaction baseline following the conclusion of the interaction (15 to 40 min). In contrast, mothers with PPD displayed a trajectory distinct from their counterparts in the non-PPD group. Their OT was observed to increase at a slower rate (more gradual increase than that shown by mothers without PPD) during interaction with their infants, failed to show an expected decrease upon conclusion of the interaction, and continued to display elevated levels even at 40 min (i.e., 25 min following the conclusion of the interaction), reflecting a potential dysregulation of the OT system as it relates to interactions with their infants. While differences in VP trajectories were less clear, both PPD and non-PPD groups showed a generally downward trend during interactions with their infants, followed by a return back to the pre-interaction baseline for the non-PPD group, in contrast to the PPD group who continued to display decreased levels of VP even at 40 min (i.e., 25 min following the conclusion of the interaction).

Our observation of a weaker temporal coupling between OT release and mother-infant interaction in the PPD group is particularly noteworthy and may reflect underlying neurobiological mechanisms contributing to impairments in mood and caregiving in PPD. Although our study was not designed to tease apart whether the observed OT dysregulation is a precursor, correlate, or consequence of PPD, this remains a compelling direction for future research. Subsequent studies should aim to further characterize the nature of OT dysregulation – specifically, whether it is best described by a delay in the timing of OT release or by a more pronounced decoupling between OT release and social behavior. Additionally, it will be important to investigate the contextual specificity of this dysregulation: Is it evident across all social contexts, or does it primarily manifest in attachment-related interactions? Are there moderating factors, such as stress, that exacerbate the dysregulation? Finally, identifying contributing influences—such as early attachment experiences, genetic predispositions, or other individual differences—will be essential to understanding the mechanisms driving the observed OT dysregulation in PPD.

### 4.3 PPD, OT dysregulation, and intergenerational risk

Our data further suggest that the mother’s OT system dysregulation may extend intergenerationally. Consistent with previously reported findings (47), our data showed positive associations between the mother’s and infant’s OT levels. The OT dysregulation seen in mothers with PPD was also reflected in these intergenerational associations, as mothers and infants in the non-PPD group displayed positive associations in their OT levels (47), but this expected pattern was not observed in mothers and infants in the PPD group.

### 4.4 Maternal and infant characteristics as potential moderators of OT dysregulation in PPD

Our findings are in line with a small body of work that has documented negative associations between maternal PPD and OT functions to date (30–33, 73). The more severe maternal PPD symptoms were, the less the mother’s OT levels increased during interactions with their infants. Our descriptive data take this a step further to suggest that this association was more specifically observed in mothers who were no longer breastfeeding or reported higher levels of childhood emotional abuse, whereas this pattern was not apparent in mothers who were still breastfeeding at the time of the study or reported lower levels of childhood emotional abuse. It is also noteworthy that mothers with higher attachment anxiety exhibited a paradoxical pattern where more severe PPD symptoms were associated with a greater increase in maternal OT levels during interactions with their infants. This pattern was not seen in mothers reporting lower levels of attachment anxiety. This finding aligns with what has previously been described as ‘paradoxical’ findings, in which increased OT is linked to greater dysfunction in the context of attachment disturbances (68). We note here that it is difficult to tease apart in our present sample whether these observed patterns reflect higher prevalence of breastfeeding difficulties, histories of childhood abuse, and attachment disturbances in mothers in the PPD (vs. non-PPD) group (see Lara-Cinisomo et al. (17), Sørbø et al. (95), Warfa et al. (96) for previously reported links between the three variables and PPD), or whether these three variables can be understood as potential risk factors for OT-related dysregulations in the presence of PPD. Nevertheless, our findings point to breastfeeding, emotional abuse, and attachment anxiety as important variables to be examined in relation to OT and mood-related dysregulations in postpartum mothers, and this would be a fruitful area of future investigation in larger samples that include depressed mothers showing a range of variabilities with respect to these three variables.

Our findings further illustrate that mothers in the PPD group generally reported more compromised maternal reflective functioning and postnatal attachment to their infants than mothers in the non-PPD group, as reflected in their PRFQ and MPAS scores, respectively. Our findings further suggest that this pattern of associations may be moderated by maternal OT functions, where depressed mothers who tended to show less increase in their OT levels during interactions with their infants tend to be more compromised in their reflective functioning and postnatal attachment. It is important to note that this pattern of association was only observed for mothers in the PPD group and not for mothers in the non-PPD group who generally showed greater levels of maternal reflective functioning and postnatal attachment in relation to their infants. This suggests that OT-related dysfunctions may be an important neurobiological correlate of compromised caregiving in mothers with PPD. Future studies should continue to investigate this important neurobiological correlate and elucidate implications that the observed OT-related dysregulations may have for innovating prevention and intervention strategies for PPD.

It is important to acknowledge potential moderating factors that were not systematically captured in the present study. The majority of mothers in the study held college or graduate degrees, which may have resulted in limited variability in socioeconomic status within our sample. This lack of variability may have constrained our ability to more fully examine the contribution of socioeconomic status to OT dysregulation and PPD. Although no significant differences in socioeconomic status were observed between the PPD and non-PPD groups in our study, low socioeconomic status—including factors such as limited income, lower educational attainment, and unemployment—is a well-established risk factor for the development of PPD (97, 98). Similarly, the present study did not assess other known risk factors of PPD, including sleep disturbances, elevated stress, increased caregiving demands, and postpartum social support. Future research should aim to include more socioeconomically diverse samples and systematically examine how these known psychosocial risk factors may influence OT functions and contribute to OT-related dysregulation in the context of PPD.

### 4.5 Strengths and limitations

The strengths of the study include our study design that involves serial hormone measurements of both OT and VP, measurements in both plasma and saliva, measurements in both mothers and infants, and a consideration of maternal characteristics that may moderate OT-related functions in PPD. Methodological discrepancies in the measurement of peripheral OT, particularly regarding immunoassay techniques and extraction protocols, have long been a subject of scrutiny in the field, and scientific consensus on optimal assay conditions remains elusive (75, 99). It is well documented that variability in assay type (e.g., radioimmunoassay vs. enzyme immunoassay), differences in assay sensitivity and specificity, and pre-analytic sample processing methods (e.g., extraction procedures) can significantly influence the quantification of peripheral OT levels. For example, the present study employed a radioimmunoassay with high sensitivity (0.1 pg), yielding baseline plasma OT levels of 3.21 ± 0.15 pg/mL. This contrasts modestly with levels reported in another study using a less sensitive radioimmunoassay (10–30 pg), which found baseline OT levels of 4.53 ± 1.18 pg/mL (100), and more substantially with a study using enzyme immunoassay, which reported levels of 122.44 ± 59.67 pg/mL (101). These discrepancies underscore the impact of methodological variation on OT quantification (102). While a comprehensive evaluation of assay accuracy and standardization is beyond the scope of the current study, the use of a highly sensitive and robustly validated immunoassay (103, 104) represents a methodological strength of the study. Moreover, our approach leveraging serial hormonal sampling, with analyses focused on relative changes (vs. absolute concentrations), constitutes a key strength in our interpretation of hormonal dynamics.

The primary limitation of this study is its small sample size which prevented us from performing more rigorous statistical tests. We acknowledge that our study is descriptive in nature and the conclusions drawn are intended to highlight patterns, trends, and potential areas for future research rather than to establish conclusive associations or causal relationships. Additionally, our missing data were exclusively reported in VP salivary measurements. Although imputation did not alter our overall findings, the loss of data may have affected the reliability of the temporal analysis of VP secretion dynamics, particularly given the modest sample size. Another limitation concerns the exploratory use of scatterplots and fitted regression lines to examine potential interaction effects. While this visual approach can be informative in our descriptive study, we acknowledge that it introduces a degree of subjectivity into interpretation, particularly in the absence of formal statistical testing for interactions. Apparent differences in slopes across groups may be influenced by outliers or random variability rather than reflecting meaningful physiological differences. Future studies with larger samples should incorporate formal tests of interaction effects involving the potential moderators identified in our exploratory work to more rigorously evaluate these patterns.

Despite these limitations, by providing descriptive data that systematically track temporal changes in OT and VP through repeated measurements for both PPD and non-PPD groups, the present study adds valuable preliminary data to the field’s understanding of temporal dynamics in OT and VP functions during postpartum mothers’ interactions with their infants and how this may become altered in the case of PPD.

### 4.6 Summary

In sum, the present descriptive study illustrated trajectories of maternal OT and VP levels during the mother’s interactions with the infant, OT’s and VP’s return to pre-interaction baseline levels following the interaction, and alterations of these trajectories in the presence of PPD. Our data also provided descriptive data on how depressed mothers’ OT-related dysregulations may have intergenerational implications for their infants, and identified several maternal characteristics (breastfeeding, history of emotional abuse, adulthood attachment anxiety, postnatal attachment to infant, parental reflective functioning) that may be closely related to OT- and mood-related dysregulations in PPD, pointing to fruitful directions for future inquiry.

## Data Availability

The raw data supporting the conclusions of this article will be made available by the authors, without undue reservation.
