# Supplementary material for: Trajectories of changes in oxytocin and vasopressin before, during, and after mother-infant interaction: a descriptive study of mothers and infants affected by postpartum depression
Source: Front Psychiatry. 2026 Jan 5;16:1636616. doi: 10.3389/fpsyt.2025.1636616 (PMC12813420; doi:10.3389/fpsyt.2025.1636616)
Supplement: Supplementary file 1 [file Table1.docx]

Supplementary Material

# Supplementary Statistical Methods

## Non-Parametric Repeated Measures Models for Changes in OT and VP Levels: PPD vs. Non-PPD Groups

Repeated measures models were fit to the data for maternal plasma OT and VP using a non-parametric approach based on rank-transformation. First, outcomes were ranked within each subject, and then a standard repeated measures model was fit using the rank-transformed data and a first-order autoregressive covariance structure (AR1). Due to limitations relating to model convergence, salivary measurements, for which there were only two available time points (0 and 20 min), were not evaluated using this method.

**1.2. Spearman Rank Correlations Between Breastfeeding Duration and OT Levels**

Spearman rank correlations were performed between breastfeeding duration and maternal and infant OT levels. Breastfeeding duration was recorded as an ordinal variable with a total of five categories: 1 = less than 2 weeks, 2 = 3-6 weeks, 3 = 7 weeks-3 months, 4 = 4-6 months, and 5 = currently breastfeeding.

# Supplementary Results

## Non-Parametric Repeated Measures Models for Changes in OT and VP Levels: PPD vs. Non-PPD Groups

Results from the non-parametric repeated measures models are presented in *Supplementary Table 4*. Consistent with findings reported in *Table 4*, we observed significant increases in maternal plasma OT at 5 min (*β* = 1.571, *t* = 2.130, *p* = .040), 10 min (*β* = 2.143, *t* = 3.170, *p* = .003), and 15 min (*β* = 1.714, *t* = 2.500, *p* = .017) following the onset of the interaction. However, we found no significant changes in maternal plasma VP at any timepoint, including at 15 min (*β* = -1.286, *t* = -1.720, *p* = .094), where a significant decrease had been observed when using the Wilcoxon Signed-Rank test. As anticipated given the limited sample size, neither the main effect of group (PPD vs. non-PPD) nor the interaction between group and timepoint survived statistical threshold of *p* < .05.

## Spearman Rank Correlations Between Breastfeeding Duration and Maternal OT Levels

Overall, mothers in the non-PPD group exhibited positive associations between baseline plasma and salivary OT levels and breastfeeding duration. In contrast, mothers in the PPD group showed a negative association between baseline plasma OT levels and breastfeeding duration. Baseline salivary OT levels demonstrated minimal to no relationship with breastfeeding duration for both mothers and infants in this group.

Following the onset of mother-infant interaction, mothers in the non-PPD group continued to show positive associations between OT levels and breastfeeding duration at the 5-min and 10-min timepoints. However, this relationship was no longer evident at 15-min and shifted to negative associations at 20 and 40 min. Similarly, infants in the non-PPD group exhibited a negative association between salivary OT levels and breastfeeding duration at the 20-min timepoint. In contrast, mothers and infants in the PPD group generally showed weak or negative associations between breastfeeding duration and OT levels across all timepoints, with the exception of maternal salivary OT at 20 minutes, which showed a positive association.

# Supplementary Figures and Tables

**Supplementary Table 1**

*Frequency and Percentages of Missing Data*

|  | Maternal Baseline Salivary Vasopressin | Maternal 20-min Salivary Vasopressin | Maternal 20-min Salivary Vasopressin % Change | Infant Baseline Salivary Vasopressin | Infant  20-min Salivary Vasopressin | Infant  20-min Salivary Vasopressin % Change |
| --- | --- | --- | --- | --- | --- | --- |
| Number Missing | 1 | 2 | 2 | 3 | 4 | 6 |
| Percent Missing | 8.33 | 16.67 | 16.67 | 25.00 | 33.33 | 50 |

**Supplementary Table 2**

*List of Medications Taken by Mothers at the Time of the Study Visit*

| *Non-PPD Group* | |
| --- | --- |
| 101 | Loestrin |
| 102 | None |
| 103 | Mirena-IUD |
| 104 | None |
| 105 | None |
| 106 | None |
| 107 | Zyrtec |
| *PPD Group* | |
| 301 | Loestrin, Claritin, Fluoxerine 20 mg |
| 302 | Metformin 1500 mg, Citalopram 20 mg, Deplin 15 mg, Doxycycline 50 mg, birth control, Zolpidem 5 mg (as needed) |
| 303 | Viibryd 40 mg |
| 304 | Zoloft, Advair inhaler, birth control |
| 306 | NuvaRing, Prozac 40 mg, Prilosec |

*Note.* The list includes all medications, including birth control, that each mother reported taking at the time of the study visit.

**Supplementary Table 3**

*Mann-Whitney U Test for Changes in OT and VP Levels from Baseline: PPD vs. Non-PPD Groups*

|  | Non-PPD (*n* = 7) | |  | PPD (*n* = 5) | |  |  |  |
| --- | --- | --- | --- | --- | --- | --- | --- | --- |
| Variable | Median | Mean Rank |  | Median | Mean Rank | *Z* | *r* | *p*-value |
| Oxytocin (OT) | | | | | | | | |
| Maternal Plasma |  |  |  |  |  |  |  |  |
| 5-min Plasma Oxytocin Change | 6.11 | 7.429 |  | 3.55 | 5.200 | -0.974 | -0.281 | 0.330 |
| 10-min Plasma Oxytocin Change | 13.99 | 6.714 |  | 7.56 | 6.200 | -0.162 | -0.047 | 0.871 |
| 15-min Plasma Oxytocin Change | 17.82 | 6.857 |  | 9.30 | 6.000 | -0.325 | -0.094 | 0.745 |
| 40-min Plasma Oxytocin Change | -2.68 | 5.429 |  | 16.46 | 8.000 | 1.137 | 0.328 | 0.256 |
| Maternal Saliva |  |  |  |  |  |  |  |  |
| 20-min Salivary Oxytocin Change | 52.17 | 7.571 |  | 26.17 | 5.000 | -1.137 | -0.328 | 0.256 |
| Infant Saliva |  |  |  |  |  |  |  |  |
| 20-min Salivary Oxytocin Change | 14.41 | 6.14 |  | 17.57 | 7.000 | 0.325 | 0.094 | 0.745 |
| Vasopressin (VP) | | | | | | | | |
| Maternal Plasma |  |  |  |  |  |  |  |  |
| 5-min Plasma Vasopressin Change | -3.50 | 7.143 |  | -4.89 | 5.600 | -0.650 | -0.188 | 0.516 |
| 10-min Plasma Vasopressin Change | -1.59 | 6.571 |  | 0.86 | 6.400 | 0.000 | 0.000 | 1.000 |
| 15-min Plasma Vasopressin Change | -6.07 | 6.857 |  | -2.31 | 6.000 | -0.325 | -0.094 | 0.745 |
| 40-min Plasma Vasopressin Change | -1.83 | 7.714 |  | -6.55 | 4.800 | -1.299 | -0.375 | 0.194 |
| Maternal Saliva |  |  |  |  |  |  |  |  |
| 20-min Salivary Vasopressin Change | -5.22 | 5.857 |  | -3.26 | 7.400 | 0.650 | 0.188 | 0.516 |
| Infant Saliva |  |  |  |  |  |  |  |  |
| 20-min Salivary Vasopressin Change | -17.17 | 5.857 |  | 1.75 | 7.400 | 0.650 | 0.188 | 0.516 |

*Note.* Values represent relative change in OT and VP levels from pre-interaction baseline (i.e., 0 min). Change scores between each timepoint and baseline were calculated and divided by the baseline value, before being converted to % changes by multiplying by 100%. *r*-statistic is reported as a measure of effect size; *r* = *Z* / sqrt(*N*).

**Supplementary Table 4**

*Non-Parametric Repeated Measures Models for Changes in Maternal Plasma OT and VP Levels from Baseline: PPD vs. Non-PPD Groups*

| Effect | Coefficient | *SE* | *t* | *p*-value |
| --- | --- | --- | --- | --- |
| Oxytocin (OT) | | | | |
| Intercept | 1.857 | 0.485 | 3.830 | 0.003** |
| Time (5-min) | 1.571 | 0.738 | 2.130 | 0.040* |
| Time (10-min) | 2.143 | 0.676 | 3.170 | 0.003** |
| Time (15-min) | 1.714 | 0.687 | 2.500 | 0.017* |
| Time (40-min) | 0.286 | 0.685 | 0.420 | 0.679 |
| PPD | -0.157 | 0.751 | -0.210 | 0.838 |
| PPD*Time (5-min) | -0.371 | 1.144 | -0.320 | 0.747 |
| PPD*Time (10-min) | -0.043 | 1.048 | -0.040 | 0.968 |
| PPD*Time (15-min) | -0.014 | 1.064 | -0.010 | 0.989 |
| PPD*Time (40-min) | 1.214 | 1.061 | 1.140 | 0.259 |
| Vasopressin (VP) | | | | |
| Intercept | 3.571 | 0.524 | 6.810 | <.0001*** |
| Time (5-min) | -0.429 | 0.835 | -0.510 | 0.611 |
| Time (10-min) | -0.857 | 0.715 | -1.200 | 0.237 |
| Time (15-min) | -1.286 | 0.749 | -1.720 | 0.094 |
| Time (40-min) | -0.286 | 0.740 | -0.390 | 0.701 |
| PPD | 0.629 | 0.812 | 0.770 | 0.457 |
| PPD*Time (5-min) | -0.771 | 1.294 | -0.600 | 0.554 |
| PPD*Time (10-min) | 0.057 | 1.107 | 0.050 | 0.959 |
| PPD*Time (15-min) | -0.314 | 1.160 | -0.270 | 0.788 |
| PPD*Time (40-min) | -2.114 | 1.146 | -1.840 | 0.073 |

*Note.* Data presented here are based on maternal plasma measurements of OT and VP. Saliva measurements were excluded due to model convergence issues. Separate models were fit for oxytocin and vasopressin.

^*^*p* < .05. ^**^*p* < .01. ^***^*p* < .001.

**Supplementary Table 5**

1. *Spearman Rank Correlations Between Breastfeeding Duration and Maternal Plasma OT Levels*

|  | Baseline  (Mother) | 5 min (Mother) | 10 min (Mother) | 15 min (Mother) | 40 min (Mother) |
| --- | --- | --- | --- | --- | --- |
| Non-PPD Group | 0.408 | 0.612 | 0.408 | 0 | -0.515 |
| PPD Group | -0.300 | 0 | -0.600 | -0.800 | -0.300 |

1. *Spearman Rank Correlations Between Breastfeeding Duration and Maternal and Infant Salivary OT Levels*

|  | Baseline  (Mother) | 20 min (Mother) | Baseline (Infant) | 20 min (Infant) |
| --- | --- | --- | --- | --- |
| Non-PPD Group | 0.612 | -0.408 | 0.204 | -0.408 |
| PPD Group | -0.100 | 0.400 | -0.100 | -0.200 |
